# Small heterodimer partner interacts with NLRP3 and negatively regulates activation of the NLRP3 inflammasome

**DOI:** 10.1038/ncomms7115

**Published:** 2015-02-06

**Authors:** Chul-Su Yang, Jwa-Jin Kim, Tae Sung Kim, Phil Young Lee, Soo Yeon Kim, Hye-Mi Lee, Dong-Min Shin, Loi T. Nguyen, Moo-Seung Lee, Hyo Sun Jin, Kwang-Kyu Kim, Chul-Ho Lee, Myung Hee Kim, Sung Goo Park, Jin-Man Kim, Hueng-Sik Choi, Eun-Kyeong Jo

**Affiliations:** 1 Department of Microbiology, Chungnam National University School of Medicine, Daejeon 301-747, South Korea; 2 Infection Signaling Network Research Center, Chungnam National University School of Medicine, Daejeon 301-747, South Korea; 3 Department of Molecular and Life Science, College of Science and Technology, Hanyang University, Ansan 426-791, South Korea; 4 Department of Anatomy, College of Medicine, Konyang University, Daejeon 302-718, South Korea; 5 Medical Proteomics Research Center, Korea Research Institute of Bioscience and Biotechnology, Daejeon 305-806, South Korea; 6 Infection and Immunity Research Center, Korea Research Institute of Bioscience and Biotechnology, Daejeon 305-806, South Korea; 7 Department of Biology, College of Life Science, Daejeon University, Daejeon 300-716, South Korea; 8 Laboratory Animal Resource Center, Korea Research Institute of Bioscience and Biotechnology, Daejeon 305-806, South Korea; 9 Department of Pathology, Chungnam National University School of Medicine, Daejeon 301-747, South Korea; 10 National Creative Research Initiatives Center for Nuclear Receptor Signals and Hormone Research Center, School of Biological Sciences and Technology, Chonnam National University, Gwangju 500-757, South Korea

## Abstract

Excessive activation of the NLRP3 inflammasome results in damaging inflammation, yet the regulators of this process remain poorly defined. Herein, we show that the orphan nuclear receptor small heterodimer partner (SHP) is a negative regulator of NLRP3 inflammasome activation. NLRP3 inflammasome activation leads to an interaction between SHP and NLRP3, proteins that are both recruited to mitochondria. Overexpression of SHP competitively inhibits binding of NLRP3 to apoptosis-associated speck-like protein containing a CARD (ASC). SHP deficiency results in increased secretion of proinflammatory cytokines IL-1β and IL-18, and excessive pathologic responses typically observed in mouse models of kidney tubular necrosis and peritoneal gout. Notably, the loss of SHP results in accumulation of damaged mitochondria and a sustained interaction between NLRP3 and ASC in the endoplasmic reticulum. These data are suggestive of a role for SHP in controlling NLRP3 inflammasome activation through a mechanism involving interaction with NLRP3 and maintenance of mitochondrial homeostasis.

## Abstract (web summary)

Excessive NLRP3 inflammasome activation underlies inflammatory diseases such as gout. Here the authors show that orphan nuclear receptor small heterodimer partner protein (SHP) negatively regulates NLRP3, and its loss leads to accumulation of damaged mitochondria and gout-like immunopathology.

## Graphical abstract

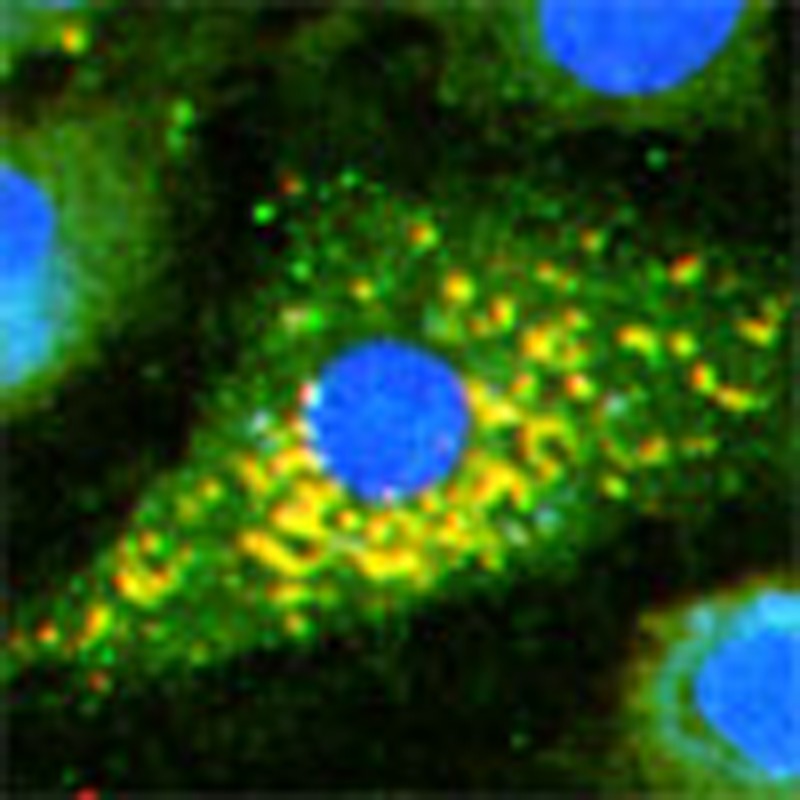

The inflammasome is a large multimeric protein complex composed of nucleotide-binding, oligomerization domain (NOD)-like receptor (NLR) proteins and adaptors that triggers caspase-1 activation, leading to maturation of the proinflammatory cytokines interleukin (IL)-1β and IL-18 (ref. 1). Among a number of inflammasomes, the NLR family, pyrin domain-containing 3 (NLRP3; also known as cryopyrin, CIAS-1, Pypaf-1 or CLR1.1) inflammasome is the best characterized. Although inflammasome activation plays a key role in host defence against a variety of pathogens, its excessive and uncontrolled activation may be damaging to the host, resulting in autoinflammatory and autoimmune diseases. It is therefore essential that inflammasome activity is tightly controlled^1^; however, the negative and counter-regulatory mechanisms of NLRP3 inflammasome activation are poorly understood.

Small heterodimer partner (SHP; also known as NR0B2) is an orphan member of the nuclear receptor (NR) superfamily. It has a unique structure that lacks the classical DNA-binding domain but contains a putative ligand-binding domain^2^,^3^. Previous work over the past 20 years has established a role for SHP as a corepressor of various genes involved in metabolic regulation, particularly those implicated in the homeostasis of glucose, bile acid and lipid metabolism^4^. However, its function in immune regulation is largely uncharacterized. Our previous work demonstrates that SHP plays a role in the regulation of Toll-like receptor (TLR)-induced innate and inflammatory responses through a biphasic interaction with cytoplasmic partners, including TRAF6 and NF-κB p65 in innate immune cells^5^,^6^.

Here we report that SHP plays a critical negative regulator of NLRP3 inflammasome activation through a physical and functional interaction with NLRP3. We found that SHP competitively inhibited the NLRP3 binding with ASC, to efficiently block the assembly of NLRP3 inflammasome complex. Using *in vivo* models of kidney tubular necrosis and peritoneal gout, we showed that SHP is essentially involved in controlling an excessive secretion of IL-1β and IL-18, as well as pathologic responses. We also showed that SHP translocated to mitochondria and dampened mitochondrial reactive oxygen species (ROS) generation and mitochondrial damage during NLRP3 inflammasome activation. Moreover, SHP deficiency led to a sustained interaction of NLRP3 with apoptosis-associated speck-like protein containing a CARD (ASC) in the endoplasmic reticulum. Our findings demonstrate that SHP plays a fine-tuning role in activation of the NLRP3 inflammasome through a direct binding with NLRP3 and elaborating mitochondrial quality control to prevent excessive inflammatory responses.

## Results

### SHP interaction with NLRP3 during inflammasome activation

**Figure 1:**
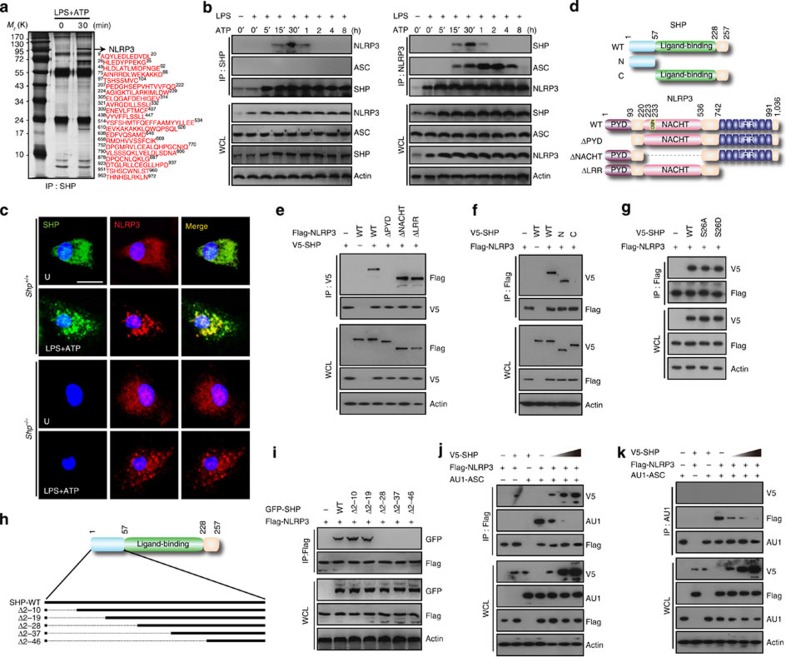
SHP interacts with NLRP3 during inflammasome activation. (**a**) Small heterodimer partner (SHP) complexes purified from lipopolysaccharide (LPS)-primed bone marrow-derived macrophages (BMDMs) with or without adenosine triphosphate (ATP, 1 mM) stimulation were subjected to mass spectrometry analysis. Red letters indicate the peptides identified. (**b**) Lipopolysaccharide (LPS)-primed BMDMs were stimulated with ATP for the indicated durations, and subjected to co-immunoprecipitation (co-IP) with antibodies for SHP (left) or NOD-like receptor family, pyrin domain-containing 3 (NLRP3; right), followed by immunoblotting (IB) with antibodies for NLRP3, apoptosis-associated speck-like protein containing a carboxy-terminal CARD (ASC), SHP and actin. (**c**) LPS-primed BMDMs from *SHP*^*+/+*^ and *SHP*^*−/−*^ mice were stimulated with ATP for 30 min, fixed, immunostained with antibodies for SHP (Alexa 488) and NLRP3 (Alexa 568), and counterstained with DAPI (blue). Upper immunofluorescence images are representative of three independent replicates; scale bar, 10 μm. (**d**) Schematic diagram of the structures of SHP (Left) and NLRP3 (Right). (**e**–**g**) 293T cells were co-transfected with a control vector, Flag-NLRP3 or truncated mutants (ΔPYD, ΔNACHT, ΔLRR), together with V5-SHP or its mutants (N-terminal or C-terminal). Cells were subjected to co-IP with antibodies for V5 (**e**) or Flag (**f**,**g**), followed by IB with antibodies for Flag or V5. (**h**) Schematic diagram of N-terminal deletions of SHP structure. ‘--’ indicates a deleted sequence. (**i**) 293T cells were co-transfected with the indicated constructs, and subjected to co-IP with anti-Flag, followed by IB analysis with antibodies for GFP or Flag. (**j**,**k**) 293T cells were co-transfected with Flag-NLRP3 or AU1-ASC, together with increasing amounts of V5-SHP, and subjected to co-IP with antibodies for Flag (**j**) or AU1 (**k**), followed by IB analysis with antibodies for Flag, AU1 or V5. Data are representative of at least three independent experiments (**a**–**c**,**e**–**g** and **i**–**k**). Protein levels in cell lysates were determined by IB analysis (**e**–**g** and **i**–**k**). U, untreated control.

To establish a role for SHP in the NLRP3 inflammasome pathway, we investigated whether SHP interacts with molecules involved in NLRP3 inflammasome activation. SHP complexes were subjected to co-immunoprecipitation (co-IP) from bone marrow-derived macrophages (BMDMs) that were primed with lipopolysaccharide (LPS) and then stimulated with adenosine triphosphate (ATP). Purified SHP complexes were then subjected to mass spectrometry analysis, which revealed that NLRP3 was the 103-kDa protein associated with SHP (Fig. 1a). Endogenous co-IP studies using an anti-SHP antibody demonstrated that SHP interacts strongly, but temporarily (from 15 min to 1 h), with endogenous NLRP3, but not with apoptosis-associated speck-like protein containing a CARD (ASC), upon NLRP3 stimulation (Fig. 1b). Furthermore, SHP co-localized with NLRP3, mainly in perinuclear regions, in LPS-primed BMDMs 30 min after ATP stimulation (Fig. 1c). Similar results, confirming an interaction between NLRP3 and SHP after NLRP3 inflammasome activation, were obtained using human THP-1 cells (Supplementary Fig. 1a,b).

Structurally, NLRP3 contains a pyrin domain (PYD) at its N terminus, a NACHT domain in the centre, and a carboxy (C)-terminal leucine-rich repeat (LRR) domain^7^. The inflammasome adaptor ASC interacts with upstream inflammasome sensor molecules through its PYD^8^,^9^. During inflammasome activation, a homotypic interaction between ASC and NLRP3 PYDs is necessary for the assembly of a large protein complex consisting of ASC multimers^10^,^11^. Further mapping studies using truncated mutants of SHP and NLRP3 (Fig. 1d) showed that the PYD of NLRP3 is required for interaction with the amino (N)-terminal portion of SHP in HEK293T cells (Fig. 1e,f). A GST pull-down assay also showed that SHP specifically and directly binds to the PYD of NLRP3 (Supplementary Fig. 2). Moreover, mutation of SHP at a site linked to SHP stability in the bile acid signalling pathway (S26A or S26D; ref. 12) was not needed for interaction of SHP with NLRP3 (Fig. 1g). We next used truncated mutants of SHP to identify the N-terminal site responsible for the interaction with NLPR3 (Fig. 1h). The SHP–NLRP3 association was detected in immunoprecipitates from two mutants (Δ2–10 and Δ2–19; Fig. 1i), but was with all other SHP N-terminal mutants (Δ2–28, Δ2–37 and Δ2–46; Fig. 1i). It is therefore likely that the 20–46 region of the SHP N-terminus is involved in PYD binding between NLRP3 and SHP.

Competition assays using SHP constructs demonstrated that increased amounts of SHP diminished the interaction between NLRP3 and ASC in HEK293T cells (Fig. 1j,k). Moreover, we treated LPS-primed BMDMs with the SHP-inducing drugs fenofibrate (a fibrate class drug for the treatment of hypercholesterolaemia^13^) and AICAR (5-amino-1-β-D-ribofuranosyl-imidazole-4-carboxamide (also known as ZMP), an analogue of AMP^14^). Both the drugs were reported to induce SHP expression through 5′ adenosine monophosphate-activated protein kinase activation^4^,^6^ and inhibited the interaction between NLRP3 and ASC in macrophages in response to NLRP3 inflammasome activation (Supplementary Fig. 3a,b). However, increased amounts of ASC did not affect the association of NLRP3 with SHP (Supplementary Fig. 4), suggesting that this association is upstream of the interaction between NLRP3 and ASC. The N terminus of the NLRP3 PYD is also required for direct binding to MAVS, another inflammasome adaptor^15^. Thus, we speculate that binding of SHP to the pyrin domain of NLRP3 reinforces the capacity of SHP to compete with ASC and MAVS.

### SHP inhibits IL-1β maturation and caspase-1 activation

**Figure 2:**
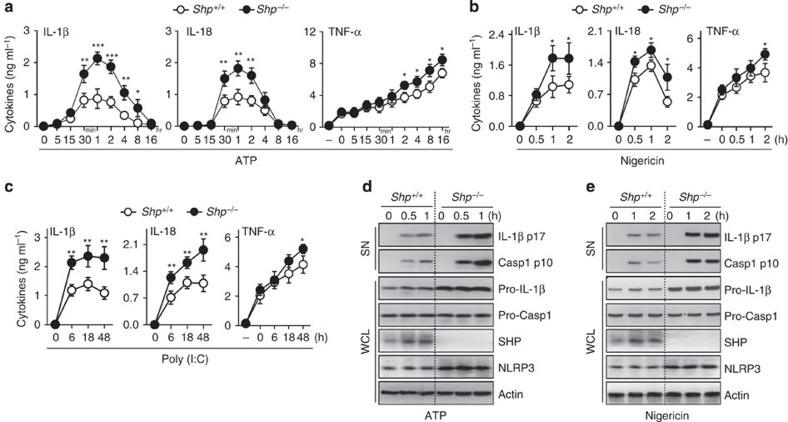
SHP deficiency increases caspase-1 cleavage and IL-1β maturation. (**a**–**e**) LPS-primed BMDMs from *SHP*^*+/+*^ and *SHP*^*−/−*^ mice were stimulated with ATP (5 mM, **a**,**d**), nigericin (15 μM, **b**,**e**), or transfected with polyinosine–polycytidylic acid (poly I:C, 5 μg ml^−1^, **c**) for the indicated durations. (**a**–**c**) Supernatants were collected and subjected to ELISA for interleukin (IL)-1β, IL-18 and tumour necrosis factor (TNF)-α. (**d**,**e**) IB analysis for IL-1β p17 or caspase-1 p10 in supernatants (SN), SHP, pro-IL-1β or pro-caspase-1 in whole-cell lysates (WCL). Actin was used as a loading control. *P<0.05; **P<0.01; ***P<0.001, compared with *SHP*^+/+^ cell cultures (two-tailed Student’s *t*-test). Data are the means±s.d. of values from four independent experiments (**a**–**c**). Data are representative of three independent experiments with similar results (**d**,**e**).

We showed previously that SHP deficiency augments the systemic inflammatory responses induced by LPS^5^. To investigate the role of SHP in regulation of inflammasome activation, BMDMs from *SHP*^*+/+*^ and *SHP*^*−/−*^ mice were stimulated with NLRP3 activators. In response to various NLRP3 inflammasome activators (ATP, monosodium urate (MSU) crystals, nigericin, poly I:C and dextran sodium sulfate), SHP-deficient BMDMs secreted higher levels of IL-1β and IL-18 than WT BMDMs (Fig. 2a–c and Supplementary Fig. 5), indicating that SHP acts as a negative regulator of NLRP3-dependent IL-1β secretion. Consistent with these results, the caspase-1 activation and IL-1β maturation observed in response to various NLRP3 inflammasome-activating stimuli were significantly increased in SHP-deficient BMDMs (Fig. 2d,e).

It is generally accepted that at least two signals (Signals 1 and 2) are required for activation of the NLRP3 inflammasome. Signal 1, a priming signal for NLRP3 inflammasome activation, is provided by pattern recognition receptors or cytokine receptors to promote NF-κB activation and increased protein expression of NLRP3. Signal 2 can be derived from danger signals, including phagolysosomal damage, mitochondrial ROS production, and induction of transmembrane ion fluxes, all of which are essential for assembly of the NLRP3 inflammasome complex^16^,^17^. We found that TNF-α levels were higher in SHP-deficient cells compared with WT cells (Fig. 2a–c and Supplementary Fig. 5a and b). Moreover, SHP deficiency led to increased induction of pro-IL-1β protein levels (Fig. 2d,e).

### SHP inhibits the Signal 2 activation of NLRP3 inflammasome

**Figure 3:**
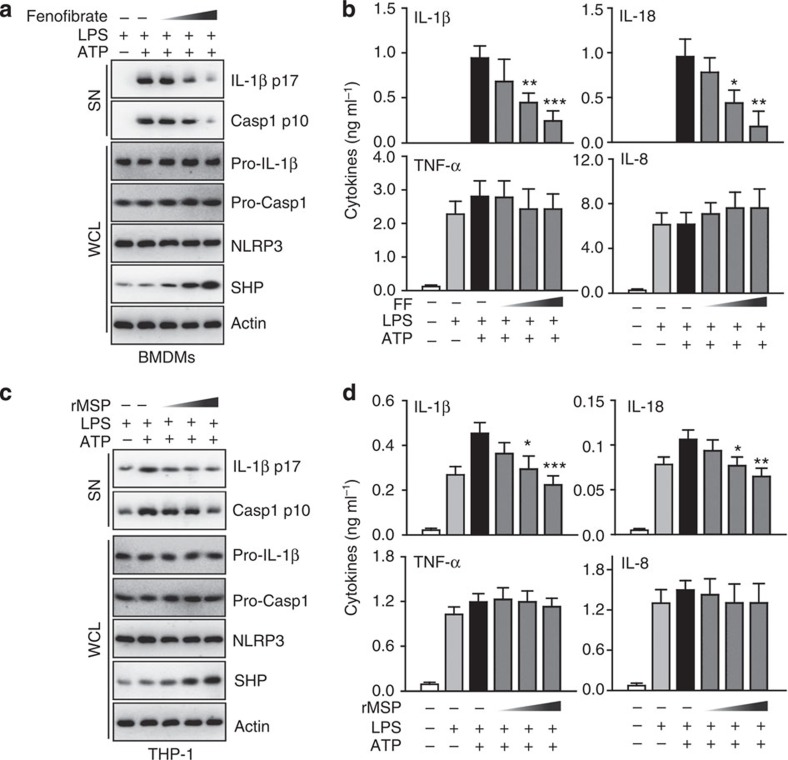
SHP inhibits the signal 2 activation of NLRP3 inflammasome. (**a**,**b**) LPS-primed BMDMs were treated with fenofibrate (10, 20, 50 μM for 4 h), or (**c**,**d**) LPS-primed THP-1 cells were treated with recombinant macrophage-stimulating protein (MSP 10, 50, 100 ng ml^−1^ for 4 h), and then activated with ATP (5 mM) for 30 min, followed by IB analysis of IL-1β p17 or caspase-1 p10 in supernatants (SN), pro-IL-1β or pro-caspase-1 in whole-cell lysates (WCL), with actin as a loading control. Data are representative of three independent experiments (**a**,**c**). Supernatants were collected and subjected to ELISA for IL-1β, IL-18, TNF-α and IL-8. Data are the means±s.d. of values from four independent experiments (**b**,**d**). *P<0.05; **P<0.01; ***P<0.001, compared with control (two-tailed Student’s *t*-test). FF, fenofibrate.

The data above suggested that a simple comparison of inflammatory cytokine production between SHP WT and KO BMDMs is not sufficient for dissecting the function of SHP in controlling Signals 1 and 2 during inflammasome activation. We thus developed other approaches to demonstrating a specific role for SHP in the regulation of Signal 2 activation of the NLRP3 inflammasome. For this, we first primed macrophages with LPS, and then treated them with a known pharmacological inducer of SHP protein (fenofibrate or recombinant macrophage-stimulating protein (MSP; the ligand of RON receptor tyrosine kinase)^4^) just before ATP or nigericin stimulation (Fig. 3 and Supplementary Fig. 3). This procedure did not affect pro-IL-1β levels or NLRP3 expression in LPS-primed cells. We next investigated whether SHP efficiently inhibited the IL-1β maturation and caspase-1 cleavage induced by ATP or nigericin stimulation. Importantly, in this experimental setting, SHP induction did not affect pro-IL-1β and NLRP3 expression levels, but abrogated IL-1β maturation and caspase-1 activation in response to NLRP3 activator treatment (Fig. 3a,c and Supplementary Fig. 3a,b). In addition, treatment of macrophages with increased amounts of fenofibrate or MSP led to dose-dependent inhibition of IL-1β and IL-18 in response to ATP stimulation, but did not attenuate TNF-α and IL-8 production (Fig. 3b,d). Together, these data provide evidence that SHP is an essential negative regulator of IL-1β maturation and caspase-1 activation in response to NLRP3 inflammasome-activating stimuli.

### SHP negatively regulates the NLRP3 inflammasome *in vivo*

**Figure 4:**
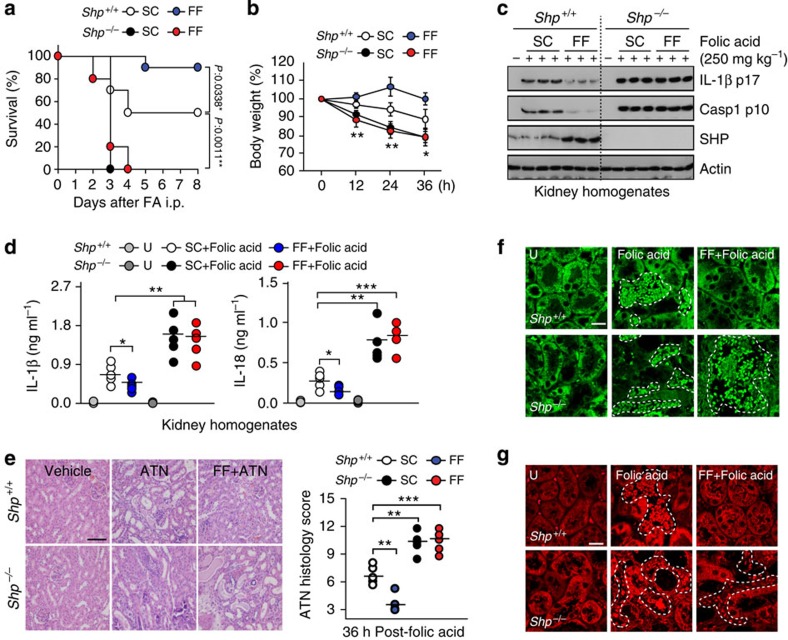
SHP deficiency exacerbates immunopathologic responses *in vivo*. (**a**–**g**) *SHP*^*+/+*^ and *SHP*^*−/−*^ mice received either fenofibrate (100 mg kg^−1^; administrated orally), or a solvent control dose, for seven consecutive days before folic acid injection (250 mg kg^−1^; *n*=10 each group, intraperitoneally (i.p.); **a**–**g**). (**a**) The survival of *SHP*^*+/+*^ and *SHP*^*−/−*^ mice was monitored for 8 days. Significant differences in comparison with the control mice are indicated (log-rank test). (**b**) Weight loss of *SHP*^*+/+*^ and *SHP*^*−/−*^ mice was monitored for the indicated durations. (**c**) IB analysis for IL-1β p17 or caspase-1 p10 from kidney homogenates. Actin was used as a loading control. (**d**) ELISA of IL-1β and IL-18 proteins in kidney homogenates. (**e**) Representative hematoxylin-eosin (H&E) stained images from kidneys at 36 h after folic acid treatment (left). ATN histology score as assessed by scoring of tubulointerstitial damage on H&E-stained kidney sections (right). Scale bar, 100 μm. (**f**,**g**) Representative immunofluorescence images of interstitial neutrophil infiltration (**f**, dotted lines) and IL-1β positive cells (**g**, dotted lines) at the corticomedullary junction of mouse kidney from each group. Scale bar, 20 μm. *P<0.05; **P<0.01; ***P<0.001, compared with control condition (two-tailed Student’s *t*-test). Data show the means±s.d. of values from five independent experiments (**b**,**c** and **e** right). Data are representative of three independent experiments with similar results (**d**,**e** left, **f** and **g**). U, untreated control; FF, fenofibrate; SC, solvent control.

Following on from the *in vitro* findings concerning NLRP3 inflammasome modulation by SHP, we investigated the regulatory role of SHP in NLRP3 inflammasome activation *in vivo*. *SHP*^*+/+*^ and *SHP*^*−/−*^ mice were subjected to folic acid-induced acute tubular necrosis (ATN)^15^. Within 4 days of folic acid injection, 50% of the *SHP*^*+/+*^ mice died (Fig. 4a). Fenofibrate-treated *SHP*^*+/+*^ mice showed significantly enhanced survival compared with solvent-treated controls. In *SHP*^*−/−*^ mice, treatment with folic acid dramatically accelerated and exacerbated morbidity, leading to death within 3 days, an effect not rescued by treatment with fenofibrate (Fig. 4a). Consistent with the survival rate data, weight loss (Fig. 4b), caspase-1 activation and IL-1β maturation (Fig. 4c), and expression of IL-1β and IL-18 (Fig. 4d) in kidney homogenates were all significantly decreased in fenofibrate-treated *SHP*^*+/+*^ mice. In *SHP*^*−/−*^ mice, however, these effects were not ameliorated by fenofibrate treatment (Fig. 4b–d).

Examination of renal pathology in mice subjected to folic acid-induced ATN revealed that, compared with *SHP*^*+/+*^ mice, *SHP*^*−/−*^ mice showed an increased incidence of pathological hallmarks, including evidence of tubular necrosis, flattening of the tubular epithelium, tubular dilatation, tubular cast formation and loss of tubular cell nuclei (Fig. 4e). Although fenofibrate treatment significantly inhibited tubulointerstitial neutrophil infiltration and IL-1β expression in WT mice, this effect could not be replicated in *SHP*^*−/−*^ mice (Fig. 4f,g). Importantly, fenofibrate treatment of *SHP*^*+/+*^ mice, but not *SHP*^*−/−*^ mice, significantly improved renal pathological responses, neutrophil infiltration and IL-1β expression (Fig. 4e–g). These results indicate that SHP is beneficial for ameliorating *in vivo* inflammatory responses induced by NLRP3 inflammasome activation.

*SHP*^*+/+*^ and *SHP*^*−/−*^ mice were also subjected to MSU crystal-induced peritonitis, a murine model of gout^18^. Six hours after *in vivo* administration of MSU crystals, IL-1β and IL-18 levels were significantly increased in cell-free peritoneal exudates from *SHP*^*−/−*^ mice, compared with those from *SHP*^*+/+*^ mice (Supplementary Fig. 6). We further examined the effect of MSP treatment on peritoneal cytokine generation in this model. MSP treatment of *SHP*^*+/+*^ mice decreased peritoneal secretion of IL-1β and IL-18, but this effect could not be replicated in *SHP*^*−/−*^ mice (Supplementary Fig. 6). These data collectively suggest a negative regulatory role for SHP in NLRP3 inflammasome activation in the context of *in vivo* inflammation models.

### SHP is required for mitochondrial translocation of NLRP3

**Figure 5:**
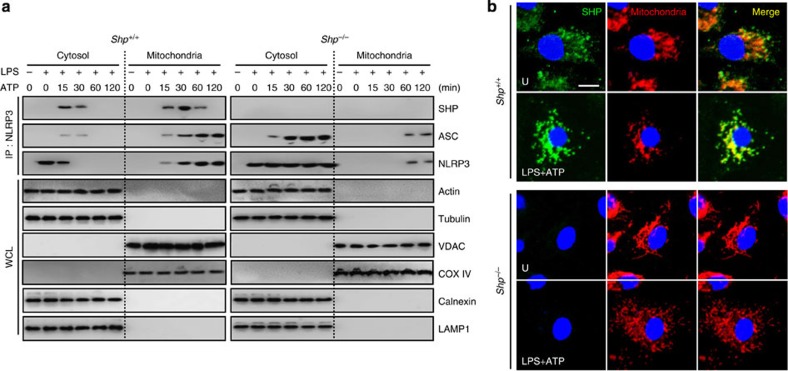
SHP is required for mitochondrial translocation of NLRP3. (**a**) BMDMs from *SHP*^*+/+*^ and *SHP*^*−/−*^ mice were primed with LPS (100 ng ml^−1^; 4 h) and stimulated with ATP (5 mM) for the indicated durations. The cells were then subcellularly fractionated, subjected to co-IP with anti-NLRP3, followed by IB analysis with antibodies for NLRP3, ASC and SHP. Levels of actin and tubulin (cytoplasm), VDAC and COX IV (mitochondria), Calnexin (ER) and LAMP1 (lysosome) protein in each fraction were determined by IB analysis. Data are representative of three independent experiments with similar results. (**b**) LPS-primed BMDMs from *SHP*^*+/+*^ and *SHP*^*−/−*^ mice were stimulated with ATP for 30 min. Co-expression of SHP (Alexa 488; green) with mitochondrial markers MitoTracker (middle) and cellular nuclei (DAPI) were visualized using immunofluorescence microscopy. Scale bar, 5 μm. Imaging data are representative of several images from three independent experiments. U, untreated control.

Assembly of the NLRP3 inflammasome requires recruitment of NLRP3 to mitochondria in the perinuclear region, leading to increased co-localization of ASC and NLRP3 (refs 15, 19, 20). It was also reported that SHP is recruited to mitochondria and regulates mitochondrial function^2^,^21^. To investigate the intracellular distribution and interaction of SHP with components of NLRP3 inflammasomes in macrophages, we performed subcellular fractionation, followed by co-IP and western blotting analysis. Notably, NLRP3 inflammasome stimulation led to the translocation of SHP and NLRP3 to the mitochondrial fraction (Fig. 5a). The analysis of confocal microscopy data also supported mitochondrial translocation of SHP after NLRP3 inflammasome activation (Fig. 5b). NLRP3 inflammasome stimulation induced sequential interaction of NLRP3 with SHP and then ASC in the mitochondrial fraction in both WT macrophages and THP-1 cells (Fig. 5a and Supplementary Fig. 7, respectively). It was also noted that SHP-deficient macrophages showed increased interaction between NLRP3 and ASC in the cytosolic fraction after NLRP3 inflammasome stimulation (Fig. 5a and Supplementary Fig. 8).

**Figure 6:**
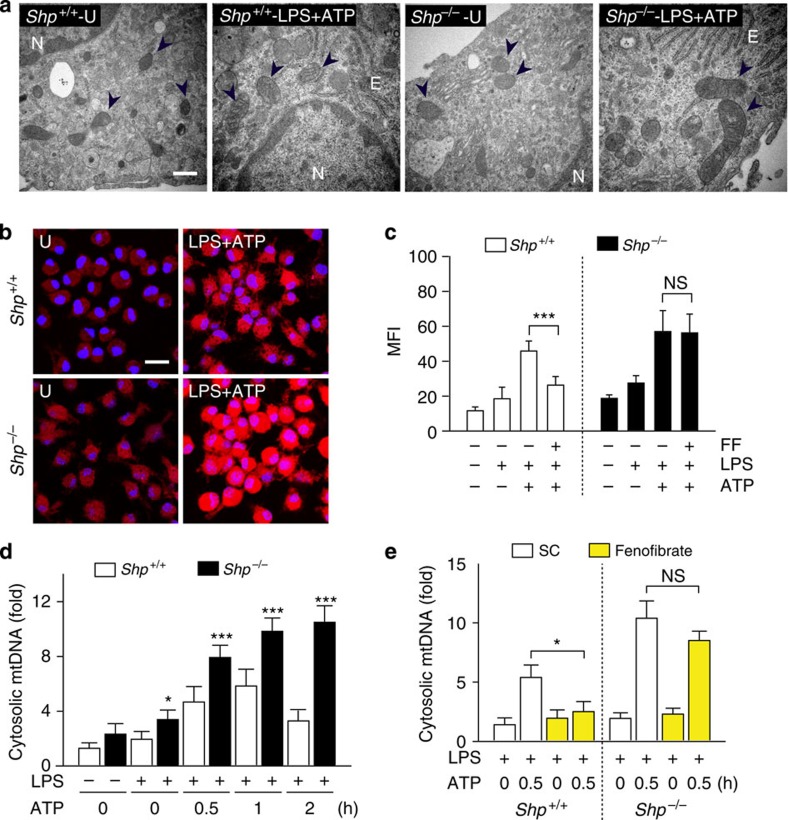
SHP is required for maintenance of mitochondrial homeostasis. (**a**) Transmission electron microscopy (TEM) analysis of BMDMs from *SHP*^*+/+*^ and *SHP*^*−/−*^ mice. Cells were unstimulated or stimulated with LPS/ATP. Black arrow, mitochondria. E, ER. N, Nucleus. Scale bar, 10 μm. (**b**) BMDMs from *SHP*^*+/+*^ and *SHP*^*−/−*^ were stimulated with or without LPS/ATP for 30 min. Cells were then labelled with MitoSOX (red) or DAPI (blue; cell nuclei) and subjected to immunofluorescence microscopic analysis for measurement of mitochondrial reactive oxygen species (ROS). Scale bar, 20 μm. (**c**) Quantitative analysis of mean fluorescence intensities (MFI) of mitochondrial ROS. LPS-primed *SHP*^*+/+*^ and *SHP*^*−/−*^ BMDMs were untreated or pretreated with fenofibrate (50 μΜ) for 1 h, and then stimulated with ATP for 30 min. (**d**,**e**) Quantitative real-time PCR analysis of cytosolic mtDNA. LPS-primed *SHP*^*+/+*^ and *SHP*^*−/−*^ BMDMs were stimulated with ATP (5 mM) for the indicated durations (**d**). LPS-primed BMDMs were untreated or treated with fenofibrate and ATP stimulation (**e**). *P<0.05; ***P<0.001, compared with control condition (two-tailed Student’s *t*-test). Imaging data are representative of several images from three independent experiments (**a**,**b**). Data are presented as the means±s.d. of values from five independent experiments (**c**–**e**). FF, fenofibrate; U, untreated control.

We next examined mitochondrial morphology and function in *SHP*^*+/+*^ and *SHP*^*−/−*^ macrophages after NLRP3 inflammasome activation. Ultrastructural findings showed that the number of swollen and dilated mitochondria with disrupted cristae was increased to a greater extent by inflammasome activation in *SHP*^*−/−*^ macrophages compared with *SHP*^*+/+*^ macrophages (Fig. 6a). In addition, LPS-primed *SHP*^*−/−*^ macrophages showed increased mitochondrial ROS generation after ATP stimulation compared with *SHP*^*+/+*^ macrophages (Fig. 6b). These data partly correlate with our previous report that SHP deficiency results in increased mitochondrial ROS generation after LPS stimulation^6^. Furthermore, fenofibrate significantly attenuated the production of mitochondrial ROS induced by inflammasome stimulation in *SHP*^*+/+*^ macrophages, but did not affect mitochondrial ROS production in *SHP*^*−/−*^ macrophages (Fig. 6c). The release of mitochondrial DNA (mtDNA) into the cytosol is dependent on mitochondrial ROS generation, and contributes to IL-1β and IL-18 secretion^22^. We therefore examined the LPS/ATP-dependent cytosolic accumulation of mtDNA in *SHP*^*+/+*^ and *SHP*^*−/−*^ macrophages. As shown in Fig. 6d, the mtDNA copy number was significantly increased in the cytosolic fraction of *SHP*^*−/−*^ macrophages, compared with *SHP*^*+/+*^ macrophages, after LPS and ATP stimulation. Moreover, the treatment of macrophages with fenofibrate markedly inhibited the accumulation of cytosolic mtDNA in *SHP*^*+/+*^ macrophages, but not in *SHP*^*−/−*^ macrophages, after NLRP3 inflammasome stimulation (Fig. 6e). These data indicate that SHP deficiency leads to accumulation of damaged mitochondria, impairs mitochondrial homeostasis, increases mitochondrial ROS generation and heightens cytoplasmic release of mitochondrial DNA in macrophages.

### The NLRP3–ASC complex localizes to ER in *SHP* ^*−/−*^ cells

**Figure 7:**
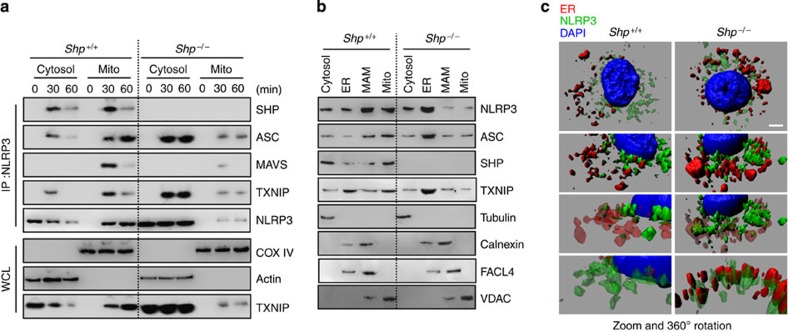
SHP deficiency induces NLRP3–ASC complex primarily localized to ER. (**a**–**c**) LPS-primed *SHP*^*+/+*^ and *SHP*^*−/−*^ BMDMs were stimulated with ATP (5 mM) for the indicated durations (**a**) or 30 min (**b**,**c**). (**a**) The cells were then subcellularly fractionated, subjected to co-IP with anti-NLRP3, followed by IB analysis with antibodies for SHP, ASC, mitochondrial antiviral-signalling protein (MAVS), thioredoxin interacting protein (TXNIP), and NLRP3. Levels of Actin (cytosolic) and cytochrome c oxidase COX IV (mitochondrial) protein in each fraction were determined by IB analysis. (**b**) Cells were subcellularly fractionated and subjected to IB analysis with antibodies for NLRP3, ASC, SHP, TXNIP. Levels of tubulin (cytosolic), calnexin (endoplasmic reticulum (ER) and mitochondria-associated membrane (MAM)), fatty acid CoA ligase 4 (FACL4, MAM) and voltage-dependent anion channels (VDAC, mitochondrial) protein in each fraction were determined by IB analysis. (**c**) ER staining and three-dimensional analysis. Data are representative of three independent experiments with similar results (**a–c**). Scale bar, 2 μm. MAM, mitochondria-associated membrane; Mito, mitochondrial fraction.

Unexpectedly, we found that mitochondrial translocation of the NLRP3–ASC complex was diminished in SHP-deficient macrophages (see Fig. 5a). To investigate this phenomenon further, we analysed the binding partners and localization of the NLRP3–ASC complex in *SHP*^*+/+*^ and *SHP*^*−/−*^ macrophages. Subcellular fractionation and co-IP analysis showed that NLRP3 was able to associate with SHP, ASC, MAVS and TXNIP in the mitochondrial fraction after NLRP3 inflammasome activation in WT macrophages (Fig. 7a). However, in *SHP*^*−/−*^ macrophages, a persistent and increased association of NLRP3 with ASC and TXNIP was observed in the cytoplasmic fractions (Fig. 7a), especially in the ER (Fig. 7b). Three-dimensional confocal microscopic analysis showed that NLRP3 was abundant in ER from *SHP*^*−/−*^ macrophages (Fig. 7c). These data suggest that SHP is required for controlled activation of the NLRP3 inflammasome complex through translocation of an SHP–NLRP3–ASC complex into mitochondria and regulation of mitochondrial homeostasis.

## Discussion

Here, we provide evidence of a novel and critical role for SHP in regulating NLRP3 inflammasome activation through physical and functional interaction with NLRP3. Several molecules, such as interferon (IFN)-γ, nitric oxide, type I IFNs and A20, have been shown to inhibit NLRP3 inflammasome activation at the transcriptional and post-translational levels^1^,^23^,^24^. We have shown here that the orphan nuclear receptor SHP inhibits assembly of the NLRP3 inflammasome and maturation of NLRP3-dependent IL-1β in the context of a variety of inflammasome-activating stimuli. In addition to its established role in regulating Signal 1 in inflammasome activation^5^, the present study provides new evidence that SHP is also involved in controlling Signal 2, triggered by NLRP3 agonists. SHP played a critical role in the inhibition of IL-1β and IL-18 production in response to various NLRP3 inflammasome-activating stimuli. Importantly, using unprimed, NLRP3-overexpressing cells, we demonstrated that SHP is critical for suppression of IL-1β and IL-18 maturation. These results implicate SHP as a key regulator of the second signalling pathway of inflammasome assembly, adding to previously discovered roles related to TLR signal regulation^5^. Around half of all mammalian NRs are able to interact with SHP. By interacting with multiple binding partners in different cells and tissues, SHP is involved in controlling a variety of biological responses, especially in metabolic and proliferative pathways^25^. Our study suggests that SHP acts as an important adaptor in innate immune activation by interacting with numerous signalling partners to efficiently block exacerbated inflammatory responses during infection or inflammation.

Furthermore, SHP is required for protection against inflammatory signal-induced pathology in several mouse models, including MSU-mediated peritonitis and acute tubular injury of the kidney. Activation of the NLRP3 inflammasome and hypersecretion of IL-1β are known to be involved in a variety of acute and chronic disorders including diabetes, atherosclerosis, gout, chronic kidney disease and Alzheimer’s disease^26^. Despite treatment advances, including several IL-1 inhibitors (rilonacept (IL-1 Trap)), canakinumab (a monoclonal anti-IL-1β antibody) and anakinra, there is an urgent need to develop novel therapeutics for the management of inflammatory diseases. We have shown that treatment with fenofibrate or MSP ameliorates the pathological changes and inflammatory cytokine production that occur in models of peritonitis (MSU-induced) and acute kidney injury by an SHP-dependent mechanism. Our data provide valuable insights for the SHP-based development of new and effective therapies targeted to pathogenic mechanisms that are mediated by NLRP3 inflammasome activation.

We have also elucidated the mechanism by which SHP fine tunes NLRP3 inflammasome responses through regulation of mitochondrial homeostasis after translocation into mitochondria as an SHP–NLRP3 complex. Mitochondrial ROS generation is an important regulatory mechanism that potentiates NLRP3 inflammasome activation in response to various stimuli^19^,^27^,^28^. Dysregulated mitochondrial activity and accumulation of ROS generating damaged mitochondria result in NLRP3 inflammasome activation^19^. Release of mtDNA into the cytoplasm and opening of the mitochondrial permeability transition pore are proposed to promote IL-1β secretion in macrophages^22^. In addition, NLRP3 Signal 2 activators can trigger the release of oxidized mtDNA into the cytosol, where it binds to the NLRP3 inflammasome to induce IL-1β production^28^,^29^. More recent studies using drugs that target mitochondria showed that NLRP3 inflammasome activation and IL-1β secretion are not only dependent on mitochondrial ROS generation but also sensitive to overall mitochondrial functional disturbance^30^. Moreover, we previously showed that the SHP-inducing, anti-lipidemic drug fenofibrate inhibits systemic inflammation through the induction of mitochondrial uncoupling protein 2 and suppression of mitochondrial ROS generation in macrophages^6^. Together, our data strongly suggest that SHP plays an important role in the fine control of mitochondrial health to prevent excessive mitochondrial damage during NLRP3 inflammasome activation.

Our data clearly show that the NLRP3 inflammasome complex localizes to the ER when damaged mitochondria accumulate in SHP-deficient macrophages. Numerous findings suggest that ER dynamics play a pivotal role in the regulation of NLRP3 inflammasome activation^31^,^32^,^33^. Indeed, NLRP3 inflammasome activation occurs at the mitochondria-associated membrane (MAM) structure^19^,^20^, the interface between the ER and the mitochondrion, where ER protein synthesis is coupled to mitochondrial metabolism^34^. In SHP-deficient cells, the distinct spatial and functional relationship between the two organelles appears to be defective, presumably owing to excessive mitochondrial damage and impaired mitochondrial homeostasis. These data indicate that SHP is critically involved in the spatial and functional coordination of NLRP3 inflammasome activation.

In conclusion, our data clearly indicate that SHP acts as a novel negative regulator of NLRP3 inflammasome activation through direct interaction with NLRP3 and fine tuning of mitochondrial quality control (see Supplementary Fig 9 for a proposed model). Our data also suggest that SHP-targeting agents ameliorate excessive NLRP3 activation, making them promising candidates for the treatment of human inflammatory diseases.

## Methods

### Mice and cell culture

Mice used in individual experiments were age- and sex-matched (6–8 weeks of age). Wild-type C57BL/6 mice were purchased from Samtako Bio Korea (Gyeonggi-do, Korea), and *SHP*^*−/−*^ mice with a C57BL/6 genetic background^35^ were kindly provided by Dr D. D. Moore (Baylor College of Medicine). The genotyping of animals was confirmed by PCR analysis. Briefly, primers used to detect the KO allele were sense, Gal-PCR 5 (5′- CTAGCTAGAGGATCCCCGGGTACC -3′) and antisense, Gal-PCR 3 (5′- AATTCGCGTCTGGCCTTCCTGTAG -3′), located in the β-gal cassette. Primers used to detect the wild-type allele were sense, Exon-1F (5′- CTCTGCAGGTC GTCCGACTATTCTG -3′) and antisense, Exon-1B (5′- CCTCGAAGGTCACAGCATCCTG -3′), located in the deleted first exon of the *SHP* gene coding region^35^. Mice were maintained under specific pathogen-free conditions.

Primary BMDMs were isolated and cultured for 5–7 days in differentiation medium containing macrophage colony-stimulating factor (25 ng ml^−1^; R&D Systems 416-ML), as described previously^36^. The culture medium was Dulbecco’s modified Eagle’s medium (DMEM; Life Technologies) containing 4 mM glutamine and 10% fetal bovine serum (FBS; Life Technologies). All animal-related procedures were reviewed and approved by the Institutional Animal Care and Use Committee, Chungnam National University College of Medicine (Daejeon, Korea).

HEK293T cells (ATCC-11268; American Type Culture Collection) were maintained in DMEM containing 10% FBS, sodium pyruvate, nonessential amino acids, penicillin G (100 IU ml^−1^), and streptomycin (100 μg ml^−1^). Human monocytic THP-1 (ATCC TIB-202) cells were grown in RPMI 1640/GlutaMAX supplemented with 10% FBS, and then treated with 20 nM phorbol myristate acetate (PMA; Sigma-Aldrich) for 24 h to induce differentiation into macrophage-like cells, followed by three washes with phosphate-buffered saline (PBS). Transient transfection was performed using Lipofectamine 2000 (Life Technologies), according to the manufacturer’s instructions.

### Reagents

Monosodium urate (MSU) crystals (tlrl-msu) and ultrapure lipopolysaccharide (LPS; tlrl-3pelps) were purchased from InvivoGen. ATP (A5394), nigericin (N7143), polyinosine–polycytidylic acid (poly I:C, P1530), folic acid (F7876), fenofibrate (F6020) and phorbol-12-myristate-13-acetate (PMA, P8139) were purchased from Sigma. Macrophage-stimulating protein (MSP) (352-MS, 6244-MS) was purchased from R&D Systems. Dextran sulfate sodium (DSS) salt (02160110) was purchased from MP Biomedicals. Dimethyl sulfoxide (DMSO; Sigma) was added to the cultures at 0.1% (v/v) as a solvent control. MitoSOX and Alexa Fluor-conjugated secondary antibodies were purchased from Life Technologies.

### Plasmid construction

DNA fragments corresponding to the coding sequences of the human NLRP3, SHP and ASC genes were amplified by polymerase chain reaction (PCR). V5-tagged SHP, Flag-tagged NLRP3 and AU1-tagged ASC were cloned into the *Xba*I and *BamH*I sites in pCDH-CMV. Flag-tagged truncated mutant constructs of NLRP3 or V5-tagged truncated mutant constructs of SHP were created by subcloning the PCR products of complementary DNA fragments, containing each domain of the target genes, into pCDH-CMV. C-terminal HA-tagged SHP WT and deletion mutants (Δ2–10, Δ2–19, Δ2–28, Δ2–37 and Δ2–46) were cloned into pEGFP-C1. All the constructs were sequenced using an ABI PRISM 377 automatic DNA sequencer to verify 100% correspondence with the original sequence.

### Construction of the expression plasmid

For the GST pull-down assay, a bicistronic expression plasmid (pIRES-SHP_1-257_-PYD_3-97_-STrEP-C_term_) was constructed for simultaneous translation of SHP and PYD genes from the same mRNA transcript. The gene encoding SHP_1-257_ was amplified by polymerase chain reaction (PCR) using primers carrying *Nhe*I and *Xho*I restriction enzyme sites at their 5′ and 3′ ends, respectively: forward, 5′- CATAGCTAGCATGGGCAGCACCAGCCAAC -3′; and reverse, 5′- CCGCTCGAGTCACCTGA GCAAAAGCATG -3′. The resulting PCR product was cloned into multiple cloning site A of the pIRES vector (Clontech Laboratories Inc.). For generation of a C-terminal Strep-tagged PYD construct, the gene encoding PYD_3-97_ was intermediately cloned into the pEXPR-IBA103 vector (IBA) using primers carrying *Xba*I and *Xho*I restriction enzyme sites: forward, 5′- GCTCTAGAATGGCAAGCACCCGCTGC -3′; and reverse, 5′- CCGCTCGAGAT CTGAACCCCACTTC -3′. It was later sub-cloned into multiple cloning site B of the pIRES vector using the restriction enzymes *Xba*I and *Not*I.

### Co-expression and purification of SHP_1-257_ and PYD_3-97_

Sixty plates (1 × 10^7^ cells per plate) were grown at 37 °C in 5% CO_2_ in a humidified incubator in Dulbecco’s modified Eagle’s medium (HyClone) supplemented with 10% fetal bovine serum, 2 mM L-glutamine, 100 U ml^−1^ penicillin and 100 mg ml^−1^ streptomycin. At 70% confluence, transient transfection was carried out with X-tremeGENE HP DNA Transfection Reagent (Roche) according to the manufacturer’s instructions. For overexpression of SHP_1-257_ and PYD_3-97_ in the HEK293T cells, cells were transfected with 15 μg of pIRES-SHP_1-257_-PYD_3-97_-STrEP-C_term_ per plate. The cells were then collected at 30 h post transfection and lysed at 4 °C in a buffer containing 50 mM Tris-HCl (pH 7.4), 7.5% glycerol, 150 mM NaCl, 1 mM EDTA, 0.1% NP-40, 1.0% sodium deoxycholate, 0.25 mM sodium pyrophosphate, 2.0 mM sodium vanadate, 2.0 mM sodium fluoride, 10 μg ml^−1^ aprotinin, 1.0 μg ml^−1^ leupeptin, 1.0 μg ml^−1^ pepstatin and 200 mM phenylmethylsulfonyl fluoride. Extracts were further incubated with avidin to mask intracellular biotin, and were then collected and cleared by centrifugation at 18,000 *g*. Before purification, co-expression of SHP and C-terminal Strep-tagged PYD was confirmed by western blotting using a rabbit polyclonal antibody against SHP (Santa Cruz Biotechnology, Inc.) or a mouse monoclonal antibody against Strep-tag (StrepMAB-Classic) (IBA). Recombinant Strep-tagged proteins were purified by affinity chromatography on a matrix carrying engineered streptavidin (Strep-Tactin) (IBA). The proteins were eluted in a buffer containing 100 mM Tris-HCl (pH 8.0), 150 mM NaCl, 1 mM EDTA and 2 mM Biotin. The eluted proteins were assessed by sodium dodecyl sulfate–polyacrylamide gel electrophoresis (SDS–PAGE).

### Adenovirus construction

Both an adenovirus encoding full-length human SHP and an SHP-specific siRNA adenovirus were constructed, as described previously^37^. Briefly, the complementary DNA sequence of full-length human SHP or an siRNA sequence specific for mouse SHP (^239^ GACAGTAGCCTTCCTCAGGAA ^250^) was incorporated into the pAdTrack-CMV shuttle vector, and a recombinant vector was generated using the AdEasy adenoviral vector system. The recombinant viruses were amplified in HEK293 cells and isolated by caesium chloride density centrifugation (Sigma).

### Analysis of inflammasome activation

Human THP-1 cells and BMDMs were primed with 100 ng ml^−1^ ultrapure LPS for 4 h in serum-free medium. Stimulations with inflammasome-activating stimuli were performed in serum-free medium (Life Technologies). Cells were treated with ATP (5 mM), nigericin (15 μM), MSU crystals (200 μg ml^−1^), poly I:C (5 μg per 10^6^ cells as indicated) for the indicated times. Poly I:C was transfected into cells using Lipofectamine 2000 (Life Technologies). At the end of the stimulation, supernatants and cell lysates were collected and stored at −20 °C. For the analysis of supernatants by immunoblotting, triplet samples were pooled and analysed with standard techniques^38^,^39^.

### Immunoblotting

Immunoblotting was performed as described previously^36^. For immunoblotting, cells were lysed in RIPA buffer containing 10 mM Tris-HCl at pH 8.0, 1 mM EDTA, 140 mM NaCl, 0.1% SDS, 0.1% sodium deoxycholate, 1% Triton X-100 and a protease inhibitor cocktail (Roche). The cell suspension was incubated at 4 °C for 15 min and then centrifuged at 14,000*g* for 15 min at 4 °C. The supernatant was collected and the protein concentration was measured by BCA assay (Pierce). The polypeptides were resolved by SDS–PAGE and transferred to a polyvinylidene difluoride (PVDF) membrane (Bio-Rad). Specific antibodies to SHP (SC-15283, dilution 1:1,000), ASC (SC-22514-R, dilution 1:1,000), NLRP3 (SC-66846, 34411, dilution 1:1,000), caspase-1 p10 (SC-514, dilution 1:1,000), MAVS (SC-166583, dilution 1:2,000), TXNIP (SC-271237, dilution 1:2,000), tubulin (SC-23948, dilution 1:5,000), VDAC (SC-271237, , dilution 1:5,000), FACL4 (SC-47997, dilution 1:1,000), FLAG (SC-807, dilution 1:1,000), V5 (SC-83849, dilution 1:1,000), GFP (SC-9996, dilution 1:1,000), LAMP1 (SC-17768, dilution 1:2,000) and actin (SC-1616, dilution 1:8,000) were purchased from Santa Cruz Biotechnology. The antibody to calreticulin (D3E6, 12238) was from Cell Signaling, IL-1β (AF-401-NA, , dilution 1:2,000) was from R&D Systems and NLRP3 (AG-20B-0014, dilution 1:1,000) was from Adipogen. The antibodies to COX IV (ab16056, dilution 1:5,000) and AU1 (ab3401, dilution 1:5,000) were purchased from Abcam. Antibody binding was visualized by chemiluminescence (ECL; Millipore) and detected by a Vilber chemiluminescence analyzer (Fusion SL 4; Vilber Lourmat). Images have been cropped for presentation. Full size images of the immunoblots are provided in Supplementary Fig. 10.

### Immunoprecipitation

Cells were collected and then lysed in RIPA buffer supplemented with a complete protease inhibitor cocktail (Roche). After pre-clearing with protein A/G agarose beads for 2 h at 4 °C, whole-cell lysates were used for immunoprecipitation with the indicated antibodies. Generally, 1–2 μg of commercial antibody was added to 1 ml of cell lysate and incubated at 4 °C for 18 h. After incubation with protein A/G agarose beads for 6 h, immunoprecipitates were extensively washed with lysis buffer and eluted with SDS loading buffer by boiling for 5 min.

### Protein purification and mass spectrometry

To identify SHP-binding proteins, LPS-primed BMDMs and THP-1 cells were stimulated with ATP for 30 min, collected and lysed with NP-40 buffer (50 mM HEPES, pH 7.4, 150 mM NaCl, 1 mM EDTA, 1% (v/v) NP-40) supplemented with a complete protease inhibitor cocktail (Roche). Post-centrifuge supernatants were pre-cleared with protein A/G beads at 4 °C for 2 h. Pre-cleared lysates were subjected to immunoprecipitation with αSHP for 18 h at 4 °C. Precipitates were washed extensively with lysis buffer. Proteins bound to beads were eluted and separated on a NuPAGE 4–12% Bis-Tris gradient gel (Life Technologies). After silver staining (Life Technologies), specific protein bands were excised and analysed by ion-trap mass spectrometry at the Korea Research Institute of Bioscience and Biotechnology Mass Spectrometry facility, and amino acid sequences were determined by tandem mass spectrometry and database searches.

### Cellular fractionation

Cytosol and mitochondria were isolated from cells using a Mitochondria Fractionation Kit (Active Motif, 40015) or as described previously^6^. Cytosol, microsomes (endoplasmic reticulum, ER), mitochondria-associated membrane (MAM) fraction and pure mitochondria were isolated from cells using an Endoplasmic Reticulum Isolation Kit (Sigma, ER0100) or as described previously^40^,^41^. Subcellular fractionated proteins were lysed in buffer containing 2% SDS and boiled with 2 × reducing sample buffer for SDS–PAGE.

### Enzyme-linked immunosorbent assay

Mouse BMDMs and human THP-1 cells were treated as indicated and processed for analysis by sandwich enzyme-linked immunosorbent assay (ELISA). Cell culture supernatants and peritoneal cavity or organ homogenates were analysed for human and mouse TNF-α, human and mouse IL-1β, human IL-8 using a BD OptEIA ELISA set (BD Pharmingen) or human (KHC0181) and mouse (KMC0181) IL-18 using an ELISA kit from Life Technologies or mouse IL-8 using an ELISA kit from Mybiosource (MBS728148). All the assays were performed as recommended by the manufacturers.

### Mitochondrial DNA quantification

To enumerate mtDNA copies in cytosol, we measured the mitochondrial (mt) to nuclear (n) DNA ratio. Mitochondrial DNA was purified using by QIAamp DNA mini kit (Qiagen) or as described previously^22^. 18S ribosomal RNA was used as a marker for nDNA and cytochrome c oxidase I for mtDNA. Real-time PCR reactions were performed according to the manufacturer’'s instructions (SYBR green PCR master mix, Qiagen), and thermal cycling was performed in a Rotor Gene 6000 instrument (Qiagen). The mtDNA content was normalized to the nucleic DNA content. The following primers were used: 18S forward, 5′- TAGAGGGACAAGTGGCGTTC -3′, and reverse, 5′- CGCTGAGCCAGTCAGTGT -3′; and mouse cytochrome c oxidase I forward, 5′- GCCCCAGATATAGCATTCCC -3′, and reverse, 5′- GTTCATCCTGTTCCTGCTCC -3′.

### Immunofluorescence and confocal microscopy

Immunofluorescence analysis was performed as described previously^36^. After the appropriate treatment, cells were washed twice with PBS, fixed with 4% paraformaldehyde in PBS for 10 min, permeabilized with 0.25% Triton X-100 in PBS for 10 min and incubated with primary antibody for 18 h at 4 °C. Cells were washed to remove excess primary antibody and incubated with the appropriate fluorescently labelled secondary antibodies for 30 min at room temperature. Nuclei were stained by incubation with DAPI for 5 min. After mounting, fluorescence images were acquired using a confocal laser-scanning microscope (LSM 710; Zeiss).

Mitochondrial ROS levels were measured in cells using MitoSOX (Life Technologies) staining (5 μM for 15 min at 37 °C). Fluorescence intensity was measured using the ImageJ or Adobe Photoshop CS4 software. For co-localization analysis, images of dynamic cell interactions were recorded as vertical *z*-stacks and processed using the Imaris 7.1.1 (Bitplane), Ultraview 5.5 (PerkinElmer) and Adobe Photoshop 7 (Adobe Systems) software to generate a three-dimensional image of the cells.

### Immunohistostaining

For immunohistostaining of tissue sections, kidneys were fixed in 10% formalin and sectioned in paraffin, as previously described^6^. To examine neutrophil infiltration or IL-1β expression, 3-μm paraffin sections were deparaffinized and hydrated by serially dipping into 100–70% ethanol, distilled water and PBS. The slides were antigen retrieved in sodium citrate buffer, blocked for 20 min in 1.5% normal rabbit serum in PBS and immunostained with antibodies specific for neutrophils (NIMP-R14, ab2557) or IL-1β (H-153, SC-7884).

### Transmission electron microscopy

For transmission electron microscopy analysis, BMDMs were washed with PBS, fixed with 4% paraformaldehyde and 2% glutaraldehyde in 0.1 M sodium cacodylate buffer (pH 7.4) for 1 h, post-fixed in 1% osmium tetroxide and 0.5% potassium ferricyanide in cacodylate buffer for 1 h, embedded in resin and cured at 80 °C for 24 h. Ultrathin sections (70–80 nm) were cut using an ultramicrotome (RMC MT6000-XL), stained with uranyl acetate and lead citrate, and examined using a Tecnai G2 Spirit Twin transmission electron microscope (FEI Co.) and a JEM ARM 1300S high-voltage electron microscope (JEOL, Tokyo, Japan).

### Folic acid-induced ATN

Folic acid (250 mg kg^−1^, Sigma) or vehicle (150 mM sodium bicarbonate) was administered intraperitoneally. Animals were weighed at time 0 and at 12, 24 and 36 h after administration of folic acid. At 36 h, mice were euthanized by CO_2_ asphyxiation. One kidney was formalin fixed and processed for H&E staining, while the other was fixed in 1% paraformaldehyde and subjected to immunofluorescence staining, as described previously^15^,^42^. Interstitial neutrophil infiltration and IL-1β-positive cells were quantified in 3-μm kidney sections stained with antibodies specific for neutrophils and IL-1β. The number of interstitial cells in the corticomedullary junction was counted in a randomly selected high-power field (× 400), and at least five fields were counted per kidney. For H&E staining, formalin fixed kidneys were paraffin-embedded, processed and sectioned at 7 μm by Histoserv Inc. Tubulointerstitial damage was assessed by scoring the following standard parameters: tubular necrosis, tubular dilatation and cast formation. Scoring was as follows; involvement of 0–25% of tubules within each corticomedullary high-powered field=1; 25–50%=2; 50–75%=3; 75–100%=4. At least 10 randomly chosen, non-overlapping fields were scored in each kidney section and all the histological examinations were performed by a nephrologist in a masked manner.

### MSU-induced peritonitis

Peritonitis was induced in 6- to 10-week-old age- and sex-matched mice by intraperitoneal injection of 1 mg kg^−1^ MSU^20^,^43^. After 6 h, the peritoneal cavity was flushed with sterile PBS. The lavage fluid was centrifuged and the supernatant was concentrated using Amicon Ultra Centrifugal Filters (Millipore) and subjected to ELISA for the indicated cytokines.

### Statistical analyses

For statistical analysis, data obtained from independent experiments (means±s.d.) were analysed using a two-tailed Student’s *t*-test. Differences were deemed to be significant at a *P* value <0.05. For survival, data were graphed and analysed using the GraphPad Prism software (GraphPad Software, Inc.). Statistical significance was evaluated using a log-rank (Mantel–Cox) test.

## Author contributions

C.-S.Y., J.-J.K. and E.-K.J. designed the research; C.-S.Y., J.-J.K., T.S.K., P.Y.L., S.Y.K., H.-M.L., D.-M.S., L.T.N., M.-S.L., H.S.J. and K.-K.K. performed the research; C.-H.L., M.H.K., S.G.P., K.-K.K. and H.-S.C. contributed the reagents; C.-S.Y., M.-S.L, H.S.J., M.H.K., S.G.P., J.-M.K. and E.-K.J. analysed the data and wrote the paper.

## Additional information

**How to cite this article:** Yang, C.-S. *et al*. Small heterodimer partner interacts with NLRP3 and negatively regulates activation of the NLRP3 inflammasome. *Nat. Commun.* 6:6115 doi: 10.1038/ncomms7115 (2015).

## Supplementary Material

Supplementary Information Supplementary Figures 1-10
