# Supplementary material for: Small heterodimer partner interacts with NLRP3 and negatively regulates activation of the NLRP3 inflammasome
Source: Nat Commun. 2015 Feb 6;6:6115. doi: 10.1038/ncomms7115 (PMC4347017; doi:10.1038/ncomms7115)
Supplement: Supplementary Information — Supplementary Figures 1-10 [file ncomms7115-s1.pdf]

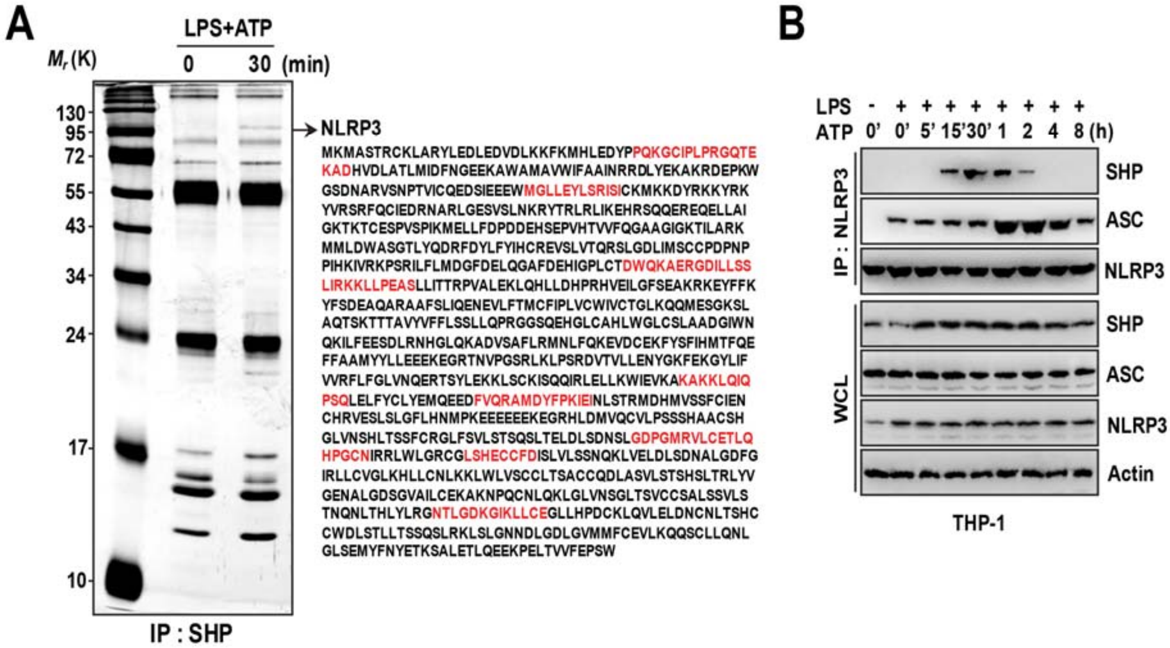


**Supplementary Figure 1. SHP interacts with NLRP3 in THP-1 cells after NLRP3 inflammasome activation.**

(**a**) Silver-stained purified of SHP complexes from LPS-primed THP-1 cells with or without ATP (1 mM) stimulation were subjected to mass spectrometry analysis. Red letters indicate the peptides identified by mass spectrometry. Data are representative of three independent experiments. (**b**) LPS-primed THP-1 cells were stimulated with ATP for the indicated times and subjected to co-immunoprecipitation (co-IP) with αNLRP3, followed by immunoblotting (IB) with αNLRP3, αASC, αSHP or αActin. Data are representative of three independent experiments.

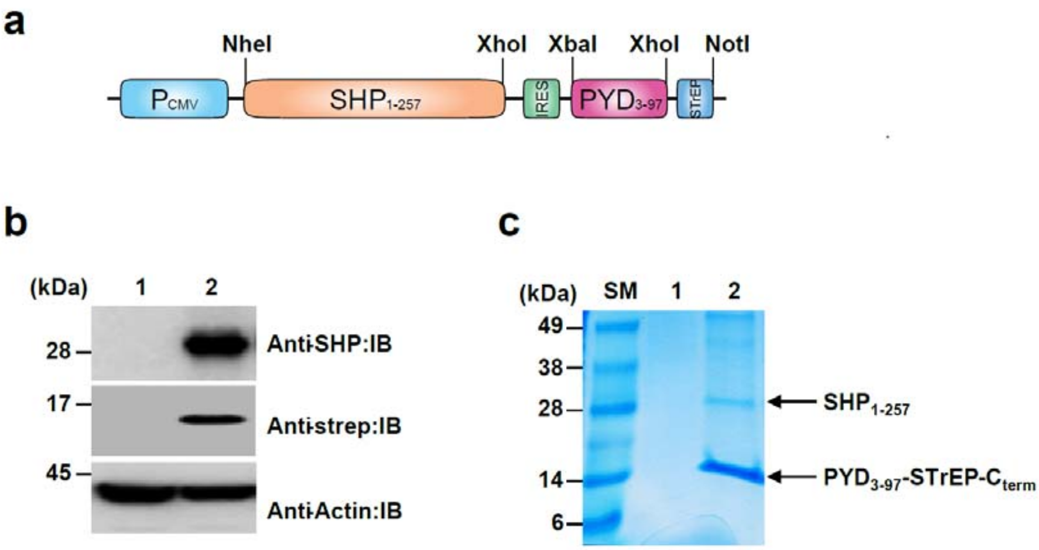


**Supplementary Figure 2. SHP directly interacts with the NLRP3 PYD domain.**

(**a**) Bicistronic plasmid containing IRES (Internal ribosome entry site) to express simultaneously $SHP_{1-257}$ and the NLRP3 $PYD_{3-97}$ domain. (**b**) Immunoblot analysis of HEK293T cells transfected with bicistronic expression plasmid pIRES-$SHP_{1-257}$-$PYD_{3-97}$-STrEP-$C_{term}$. Lane 1, lysates transfected with empty vector; lane 2, lysates with pIRES-$SHP_{1-257}$-$PYD_{3-97}$-STrEP-$C_{term}$. (**c**) Pull-down purification using the strep-tagged PYD. Purified proteins were visualized by SDS-PAGE Coomassie blue staining. SM, size marker; lane 1, empty vector; lane 2, pull-down purified $SHP_{1-257}$ and $PYD_{3-97}$-STrEP complex. Data are representative of two independent experiments

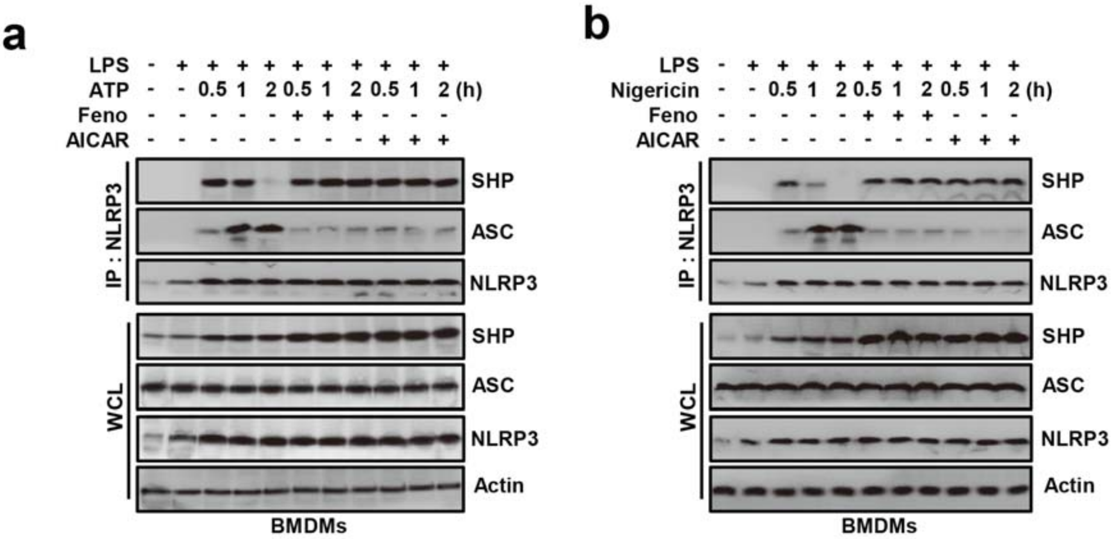


**Supplementary Figure 3. Increased amounts of SHP result in decreased interaction between NLRP3 and ASC.**

LPS-primed BMDMs were treated with the AMPK activator fenofibrate (10, 50, 100 μM for 4 h) or AICAR (0.1, 0.5, 1 mM for 4 h), and then stimulated with ATP (5 mM, **a**) or nigericin (15 μM, **b**) for the indicated times. Cells were then subjected to co-IP with αNLRP3, followed by IB analysis with αSHP, αASC, αNLRP3 or αActin. Data are representative of three independent experiments.

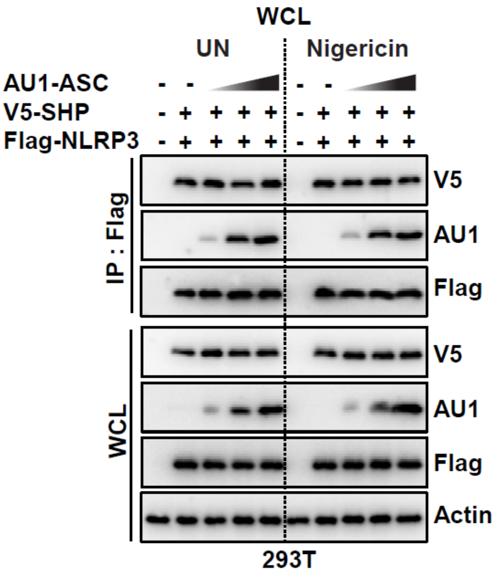


**Supplementary Figure 4. The increased amounts of ASC did not inhibit the interaction between NLRP3 and SHP.**

293T cells were co-transfected with Flag-NLRP3 or V5-SHP, together with increasing amounts of AU1-ASC for 48 h. Cells were stimulated with or without nigericin (15 μM) for 45 min. and subjected to co-IP with antibodies for Flag, followed by IB analysis with antibodies for Flag, AU1 or V5. Data are representative of at least three independent experiments

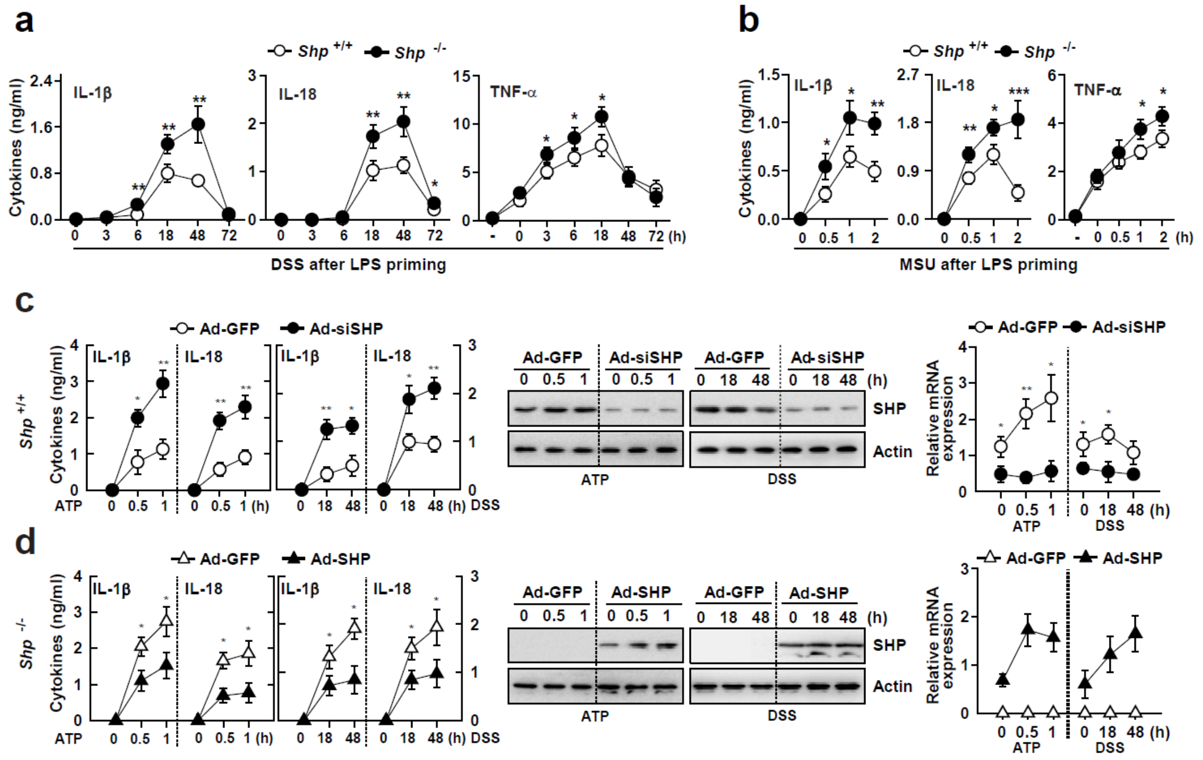


**Supplementary Figure 5. Loss of SHP enhances DSS- or MSU-induced IL-1β and IL-18 production in LPS-primed macrophages.**

LPS-primed BMDMs were stimulated with DSS (3%, **a**) or MSU (200 μg/ml, **b**) for the indicated times. (**c** and **d**) BMDMs from WT mice were transduced with Ad-GFP or Ad-siSHP (MOI = 10) or BMDMs from KO mice were transduced with Ad-GFP or Ad-SHP (MOI = 10) for 48 h, followed by primed with LPS (100 ng/mL; 4 h) prior to ATP (5 mM) or DSS (3 %) stimulation for the indicated times. (**a** and **b, c** and **d**, left) Supernatants were collected and subjected to ELISA for IL-1β, IL-18 and TNF-α. (**c** and **d**, middle) Western analysis for SHP protein expression for an adenovirus transduction efficacy. Actin was used as a loading control. (**c** and **d**, right) Quantitative real-time PCR of *Shp* for an adenovirus transduction efficacy. **P* < 0.05; ***P* < 0.01; ****P* < 0.001, compared with the control condition (two-tailed Student's *t*-test). Data are the means ± SD of values from four independent experiments.

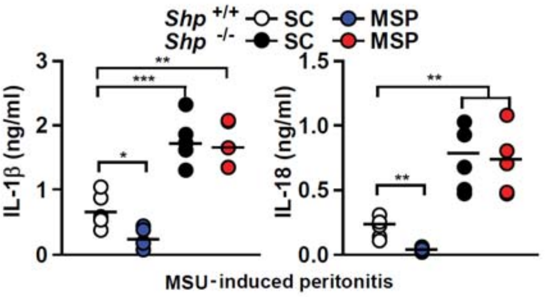


**Supplementary Figure 6. SHP deficiency exacerbates NLRP3 inflammasome activation in peritonitis.**

*SHP*$^{+/+}$ and *SHP*$^{-/-}$ mice received either MSP (500 μg/kg; i.p) or solvent control for seven consecutive days before MSU crystal injection (1 mg/kg; n = 5 each group, i.p). After 6 h, peritoneal exudates were harvested and subjected to ELISA for IL-1β and IL-18 protein levels. *$P < 0.05$; **$P < 0.01$; ***$P < 0.001$, compared with control condition (two-tailed Student's *t*-test).

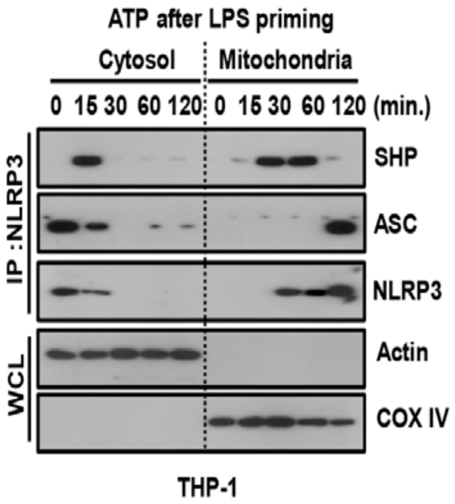


**Supplementary Figure 7. SHP interaction leads to NLRP3 recruitment of mitochondria.** LPS-primed THP-1 cells were stimulated with ATP (5 mM) for the indicated times. The cells were subcellularly fractionated and subjected to co-IP with αNLRP3, followed by IB analysis with αNLRP3, αASC, and αSHP. Levels of β-actin (cytosolic) and COX IV (mitochondrial) protein in each fraction were determined by IB analysis. Data are representative of three independent experiments with similar results.

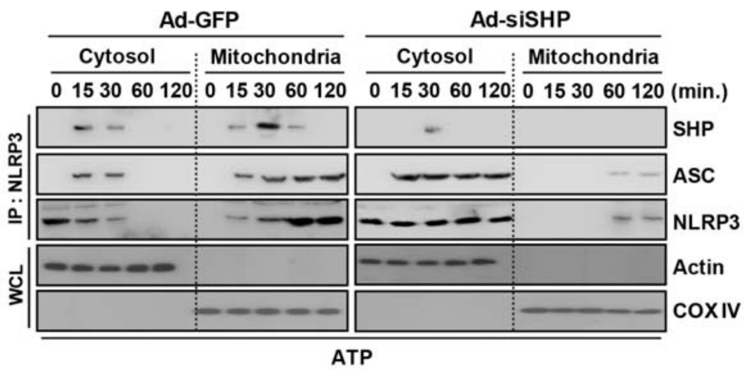


**Supplementary Figure 8. SHP is required for mitochondrial translocation of NLRP3.**
BMDMs from WT mice were transduced with Ad-GFP or Ad-siSHP (MOI = 10) for 48 h, primed with LPS (100 ng/ml; 4 hr) and stimulated with ATP (5 mM) for the indicated durations The cells were then subcellularly fractionated, subjected to co-IP with anti-NLRP3, followed by IB analysis with antibodies for NLRP3, ASC, and SHP. Levels of Actin (cytosolic) and COX IV (mitochondrial) protein in each fraction were determined by IB analysis. Data are representative of three independent experiments with similar results.

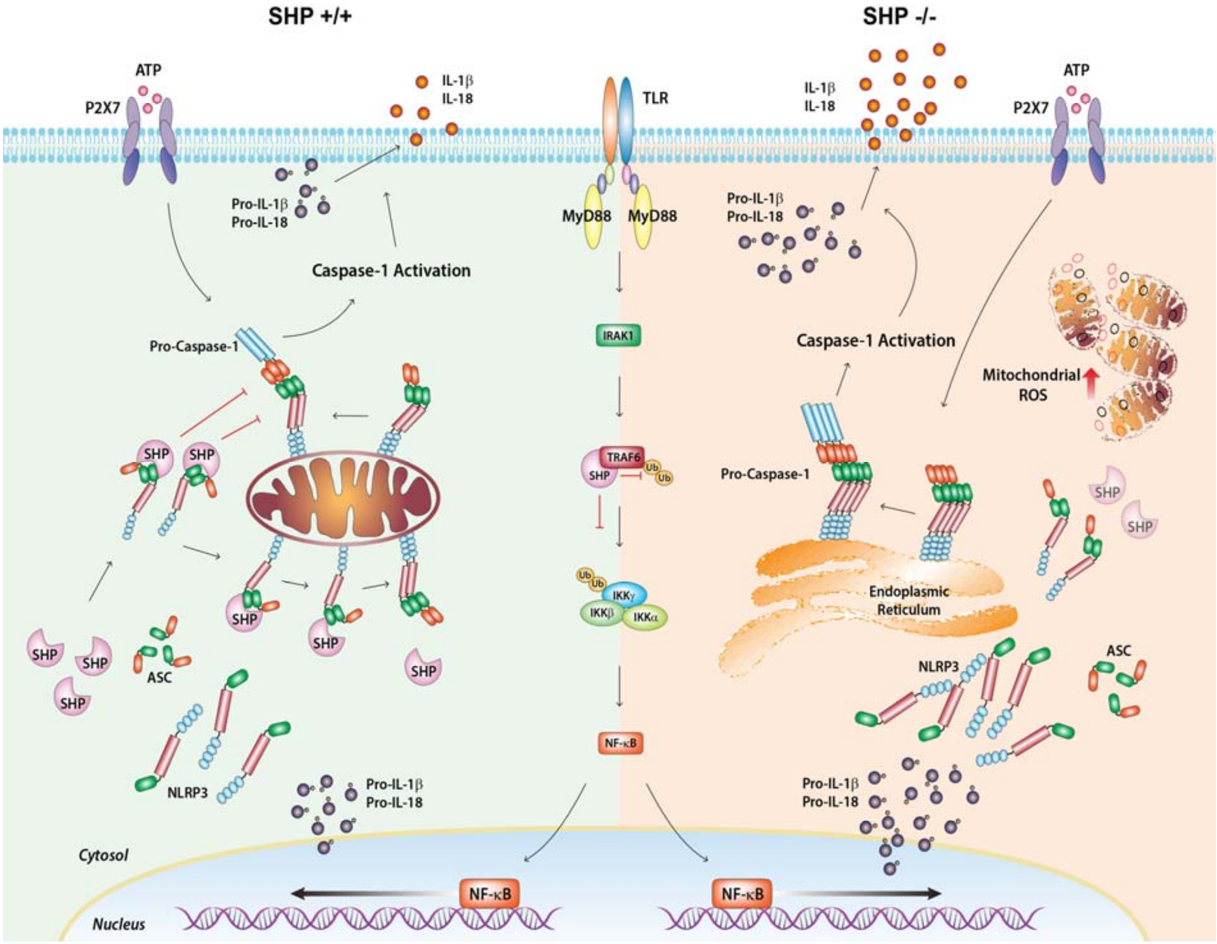


**Supplementary Figure 9. Schematic model for the roles of SHP and SHP-mediated regulatory pathways in NLRP3 inflammasome activation.**

We showed previously that SHP negatively regulates TLR signaling in macrophages by interacting with TRAF6 and inhibiting its polyubiquitination (Yuk et al., *Nat Immunol*, 2011). In the current study, we report that SHP acts as a negative regulator of NLRP3 inflammasome signaling in macrophages. During NLRP3 inflammasome activation, SHP directly interacts with NLRP3, and then both proteins are recruited to the mitochondria to maintain mitochondrial homeostasis. Increased SHP levels inhibit the interaction of NLRP3 with ASC. SHP-deficient macrophages show impaired mitochondrial quality control (in terms of damaged mitochondria accumulation, mitochondrial ROS generation, and mitochondrial DNA release) and a sustained interaction between NLRP3 and ASC in the endoplasmic reticulum.

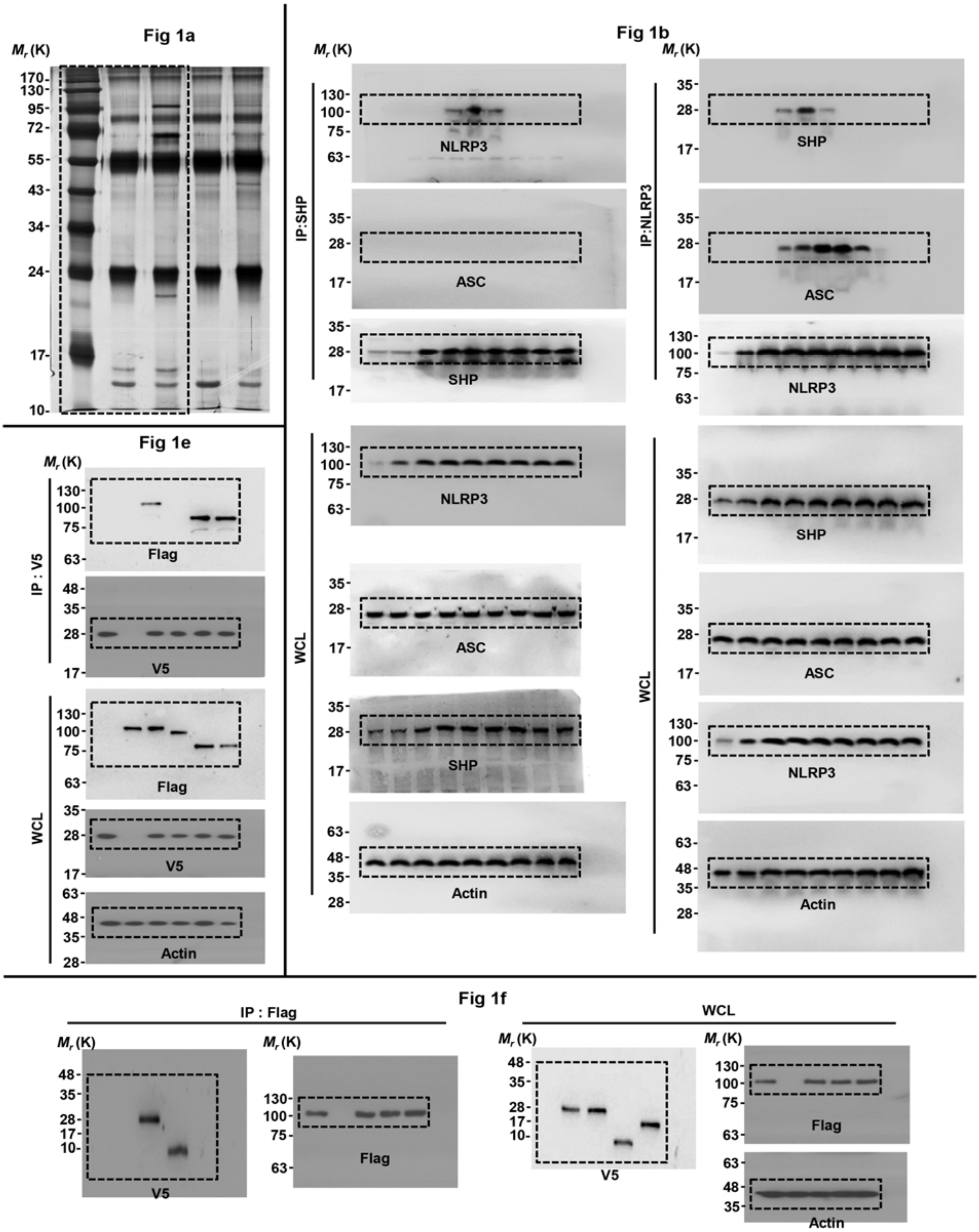


**Supplementary Figure 10.**

**Full scan image of the western blots used in the manuscript for Fig. 1a, b, e, and f**

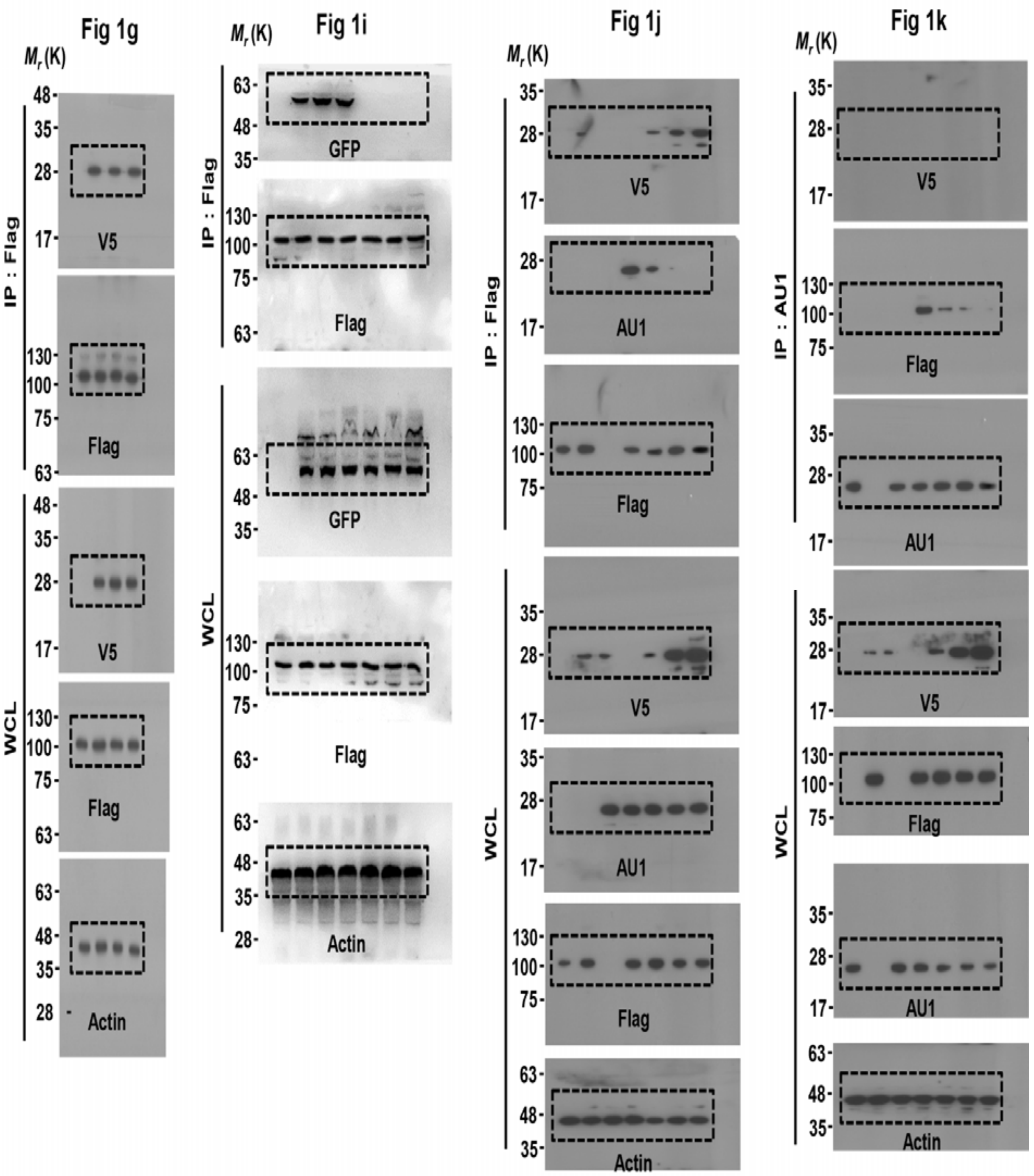


**Supplementary Figure 10 (Countined).**

**Full scan image of the western blots used in the manuscript for Fig. 1g and i-k**

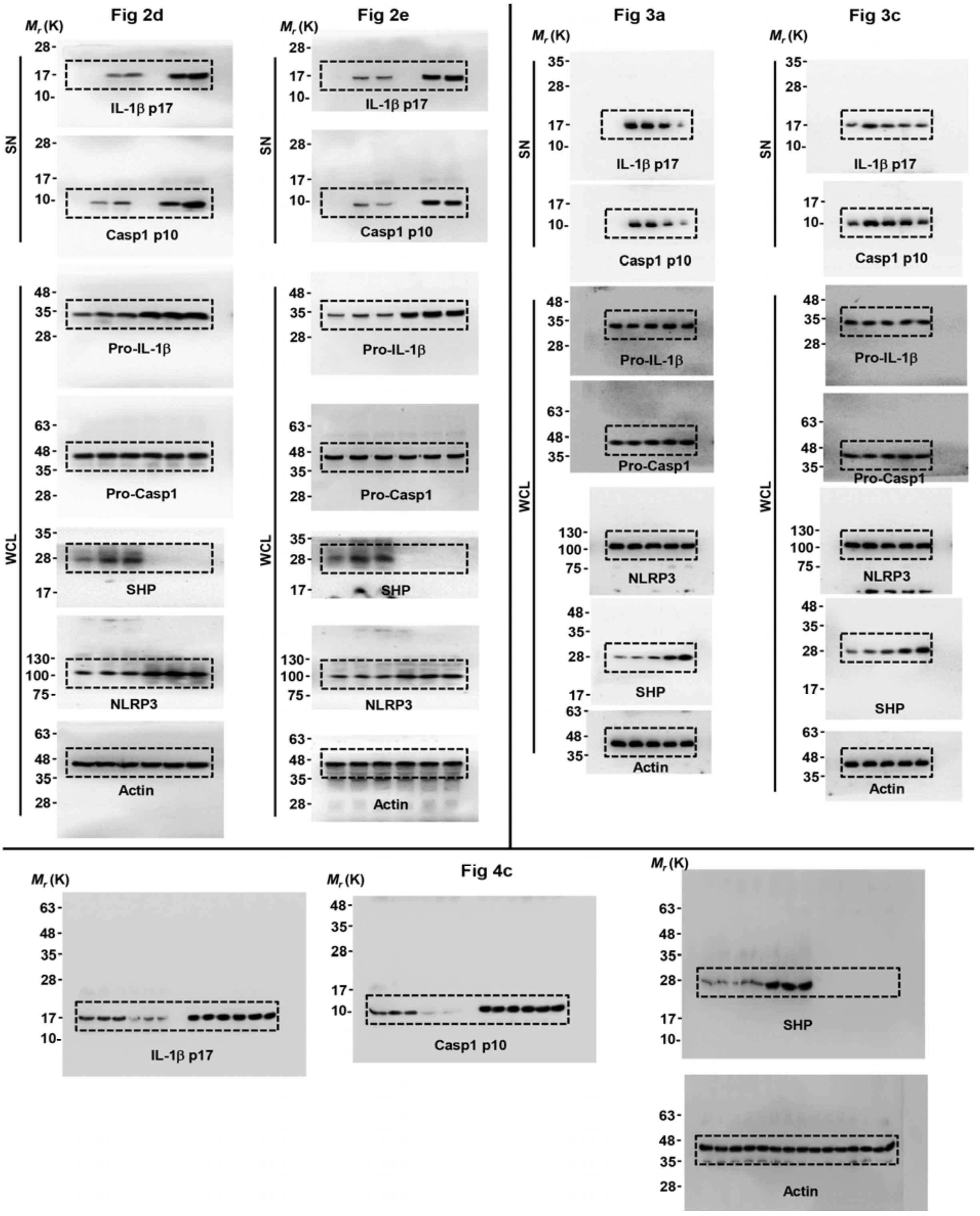


**Supplementary Figure 10 (Countined).**

**Full scan image of the western blots used in the manuscript for Fig. 2-4.**

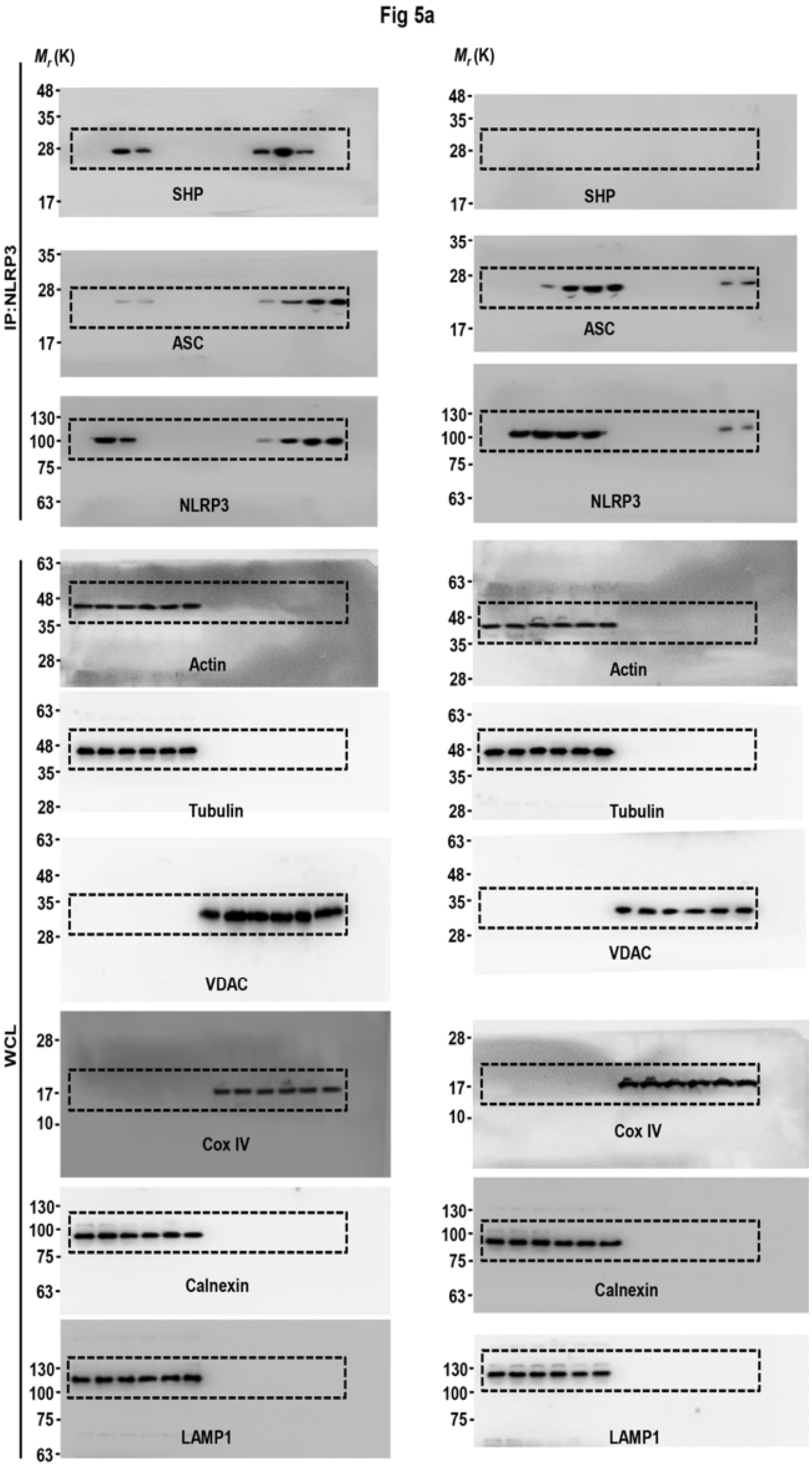


**Supplementary Figure 10 (Countined).**

**Full scan image of the western blots used in the manuscript for Fig. 5a**

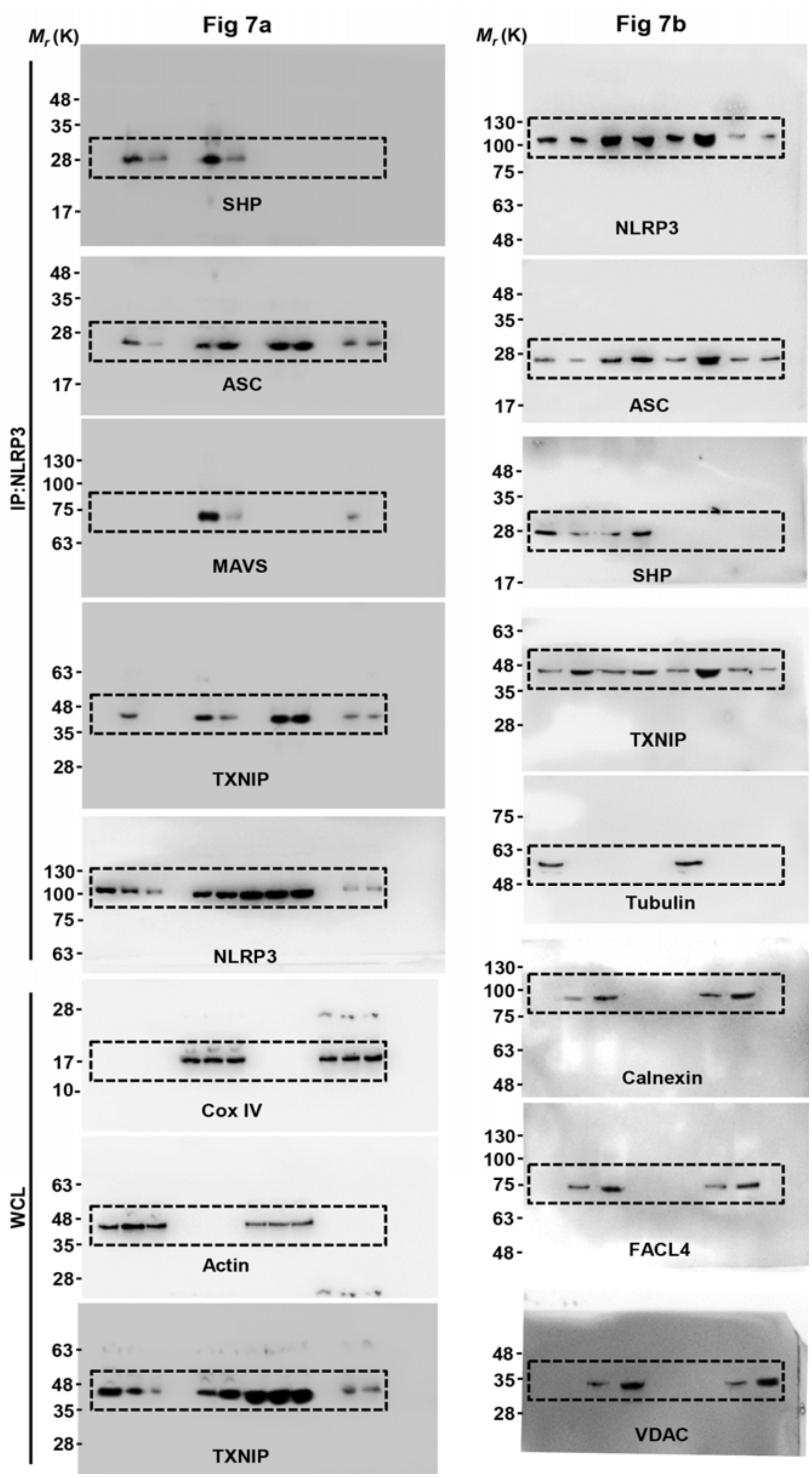


**Supplementary Figure 10 (Countined).**

**Full scan image of the western blots used in the manuscript for Fig. 7a and b.**

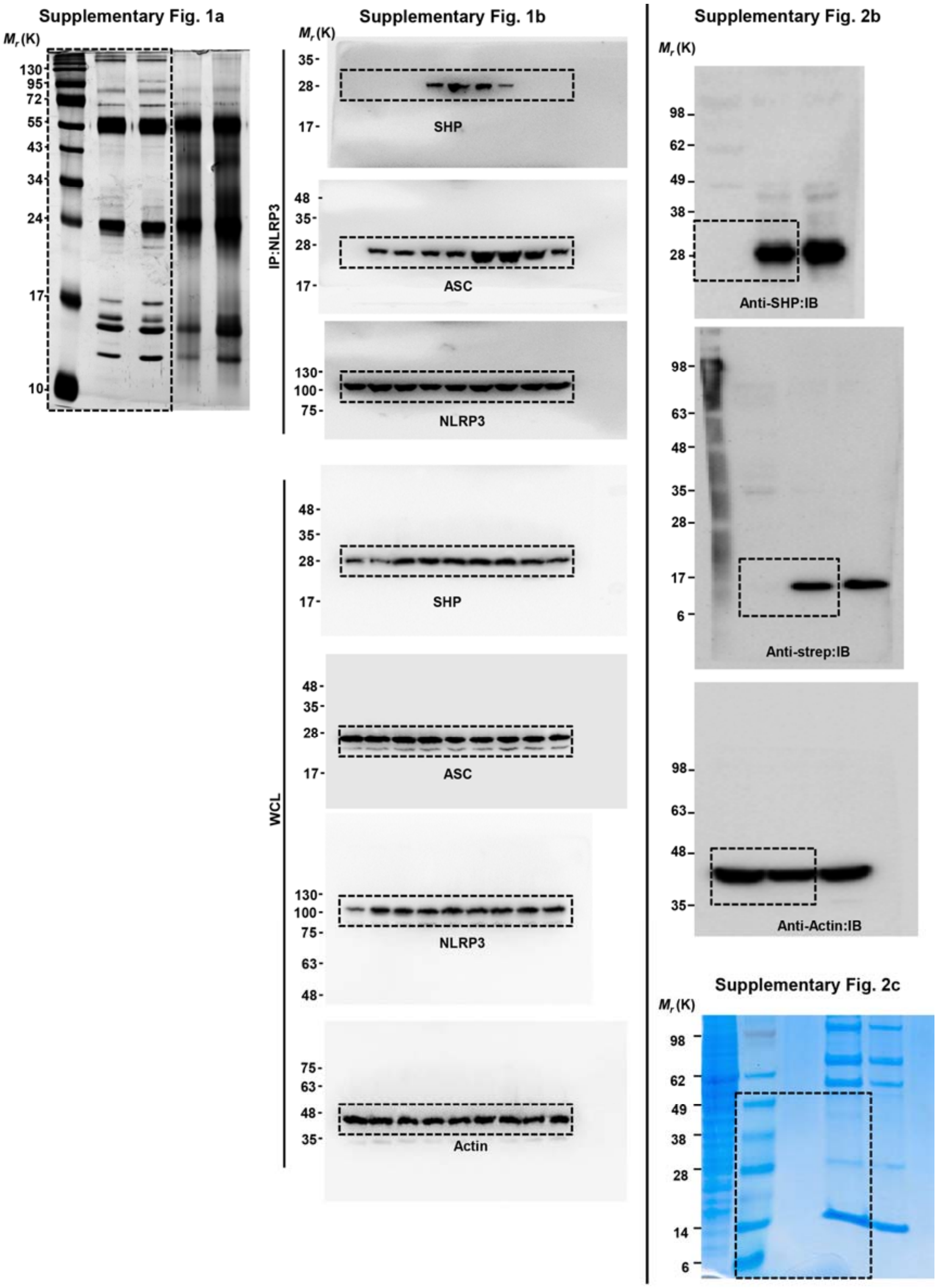


**Supplementary Figure 10 (Countined).**

**Full scan image of the western blots used in the manuscript for Supple. Fig. 1-2.**

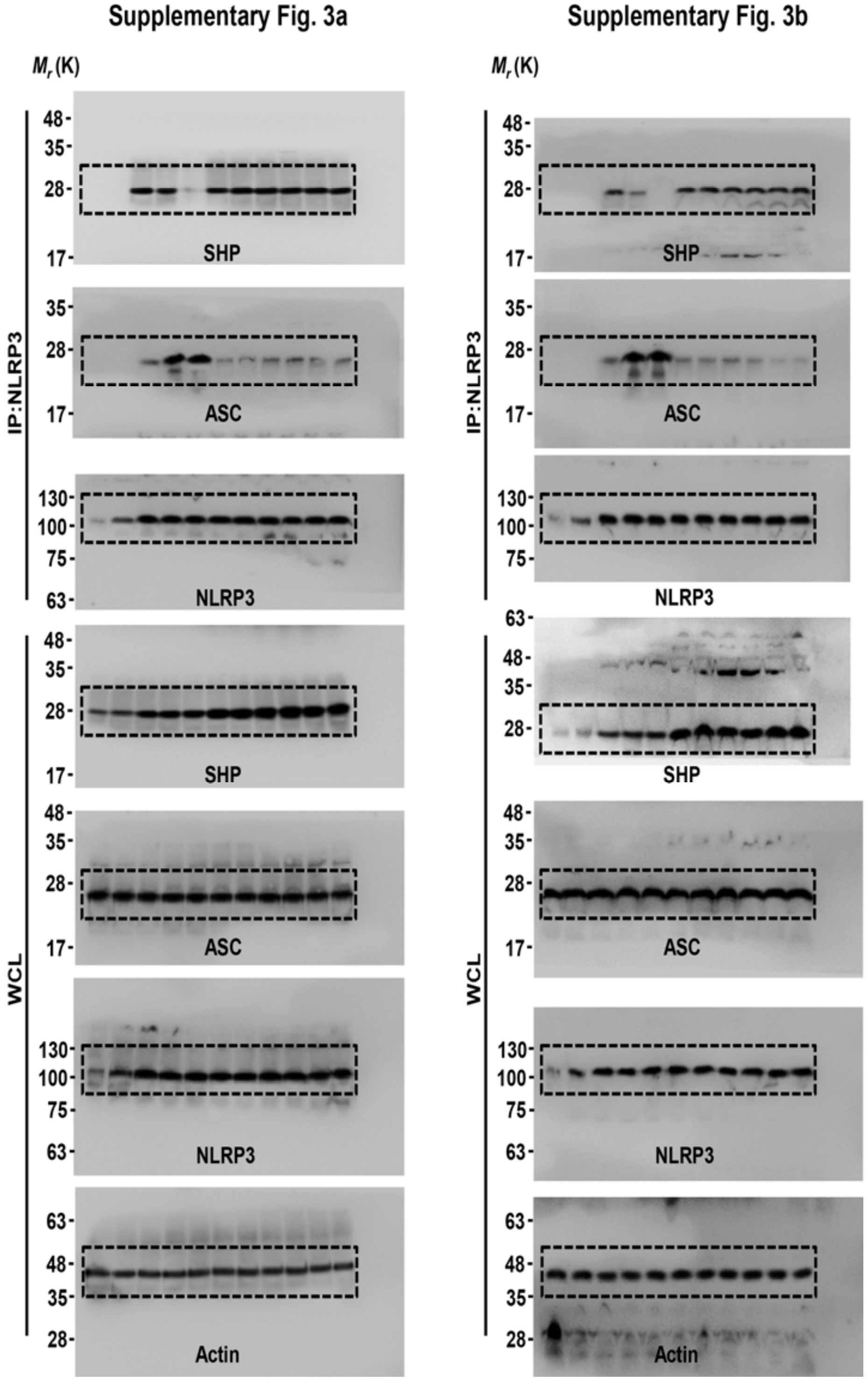


**Supplementary Figure 10 (Countined).**

**Full scan image of the western blots used in the manuscript for Supple. Fig. 3a and b.**

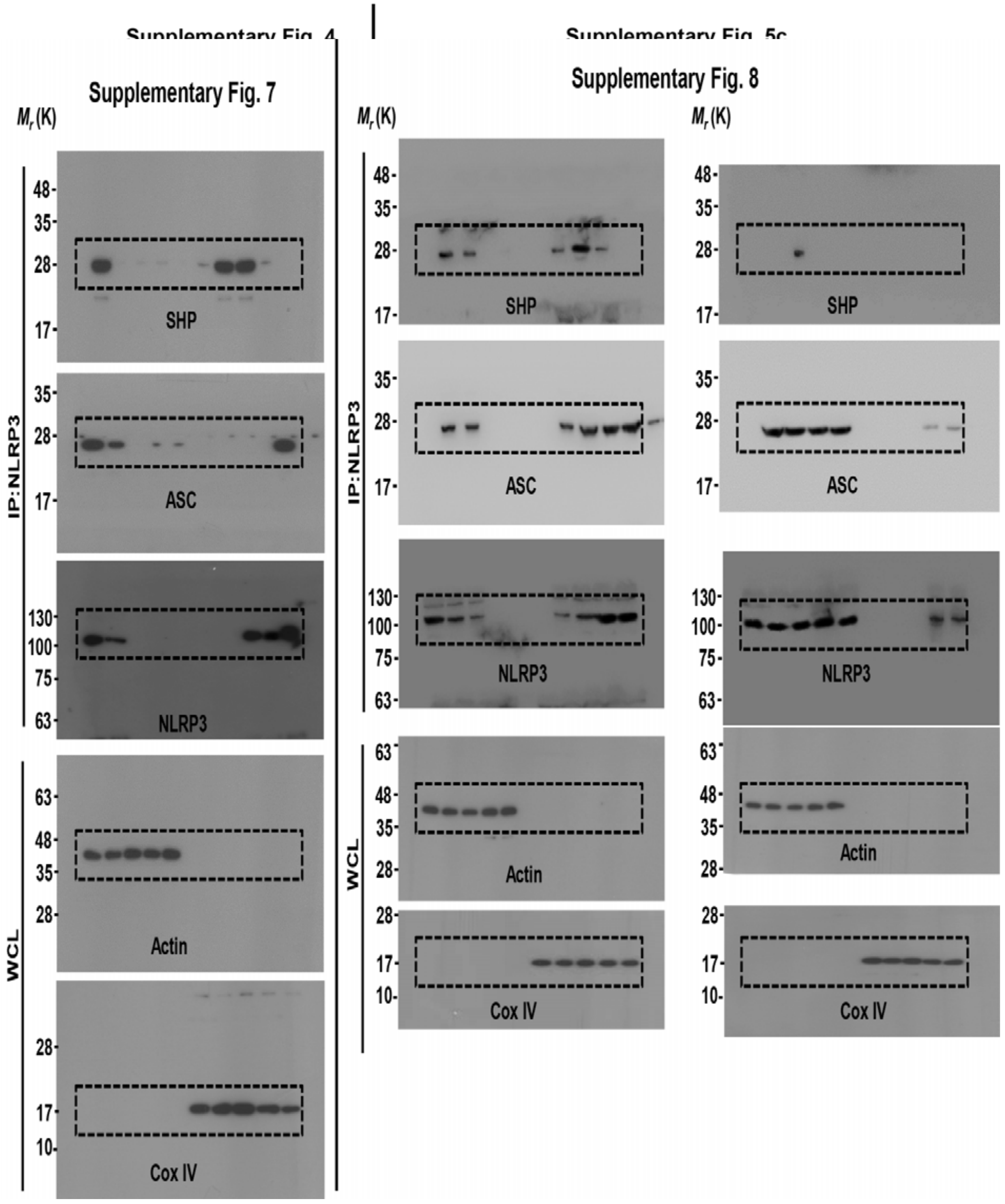


**Supplementary Figure 10 (Countined).**

**Full scan image of the western blots used in the manuscript for Supple. Fig. 4-5.**

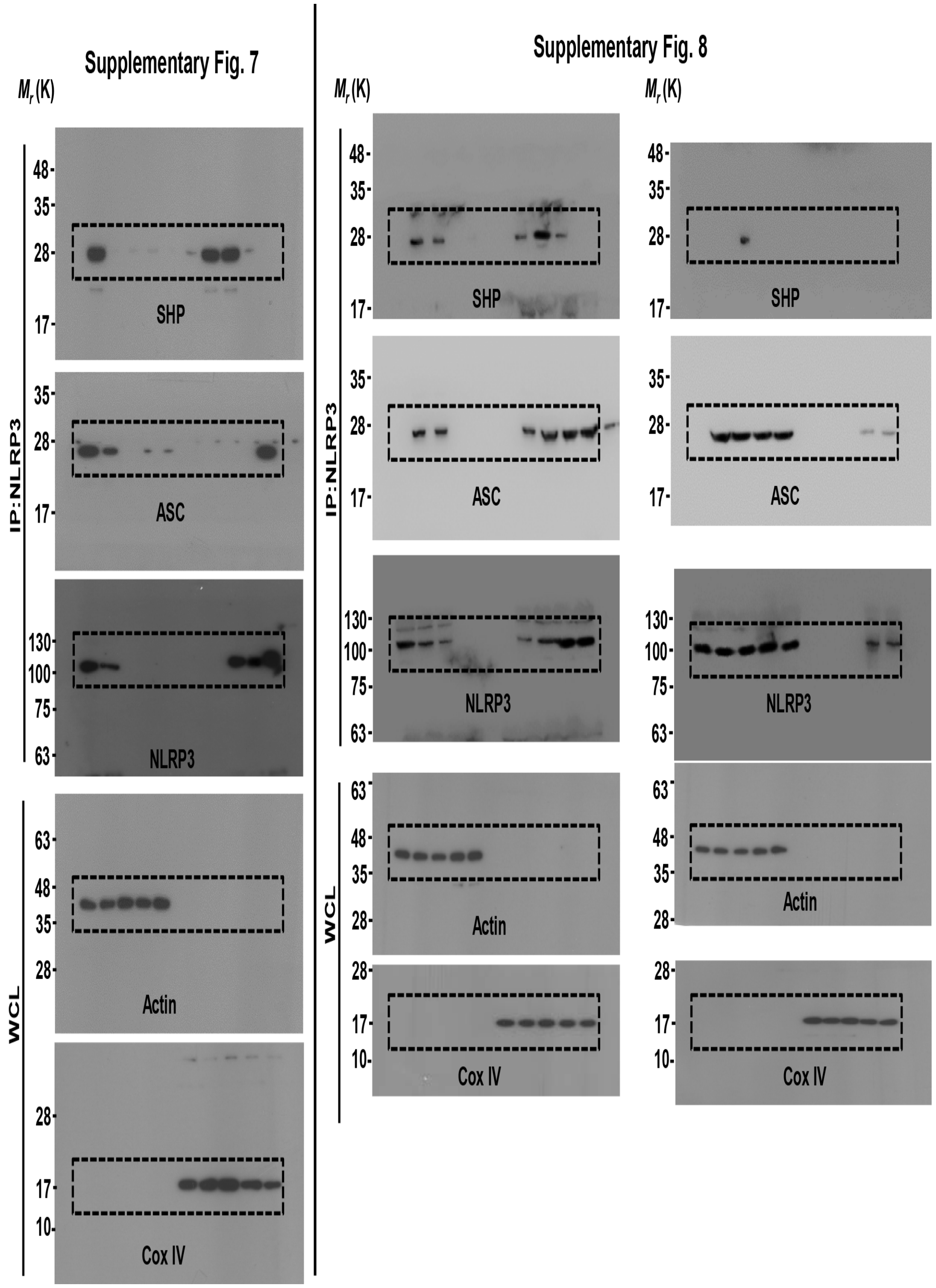


**Supplementary Figure 10 (Countined).**

**Full scan image of the western blots used in the manuscript for Supple. Fig. 7-8.**
